# Phytochemical Diversity in *Populus trichocarpa* Buds: Insights into Population Variation and Antifungal Properties

**DOI:** 10.3390/plants15111746

**Published:** 2026-06-04

**Authors:** Sam C. Cothron, Luke Leftwich, Jin-Gui Chen, Feng Chen

**Affiliations:** 1 Graduate School of Genome Science and Technology, University of Tennessee, Knoxville, TN 37996, USA; 2 The Center for Bioenergy Innovation, Oak Ridge National Laboratory, Oak Ridge, TN 37831, USA; 3 Department of Biochemistry & Cellular and Molecular Biology, University of Tennessee, Knoxville, TN 37996, USA; lleftwi2@vols.utk.edu; 4 Biosciences Division, Oak Ridge National Laboratory, Oak Ridge, TN 37831, USA; 5 Department of Plant Sciences, University of Tennessee, Knoxville, TN 37996, USA

**Keywords:** *Populus*, dormant buds, population, antifungal, terpenoids

## Abstract

Buds are a critical stage in the annual growth–dormancy cycle of perennial woody plants and are essential for survival and biomass accumulation. To safeguard these structures, trees employ both physical and chemical protection. Although *Populus* buds are known to contain rich phytochemistry, population-level variation remains largely unexplored. Here, we characterized bud phytochemistry across a population of *Populus trichocarpa* natural variants using gas chromatography–mass spectrometry and examined the antifungal properties of bud extracts. In the reference genotype Nisqually-1, a total of 32 lipophilic metabolites were detected, belonging to four chemical groups: terpenoids, phenylpropanoids, linear hydrocarbons, and others. Analysis of 49 additional *P. trichocarpa* natural variants revealed both shared features and substantial variation. All lines contained metabolites from the phenylpropanoid, linear hydrocarbon and terpenoid classes, which consistently dominated the profiles. However, quantitative differences in individual metabolites and relative class abundances distinguished the lines, allowing them to be grouped into three chemotypic clusters. To assess potential biological implications of phytochemical variance, we tested antifungal activity of bud extracts against the pathogenic fungus *Fusarium oxysporum*. Extracts from all 50 lines significantly inhibited fungal growth compared with controls. Correlation analyses between metabolite abundance and inhibition strength identified candidate metabolites that were most strongly associated with antifungal activity. Together, these findings reveal both conserved and variable components of bud phytochemistry in *P. trichocarpa*. The observed chemical diversity and consistent antifungal effects suggest that bud metabolites contribute to defense and may reflect adaptation across natural populations.

## 1. Introduction

Buds are critical plant structures, housing the shoot apical meristem and, in some cases, floral primordia, and their protection is essential for survival and reproductive success [1,2,3]. Early land plants possessed relatively exposed or “naked” buds, but in higher plants these structures are enclosed by specialized leaves, or bud scales, that form a durable physical barrier against environmental stressors, herbivores, and pathogens during periods of dormancy [4]. Bud scales are partially lignified and covered by a waxy cuticle enriched in cutins and suberins, providing substantial structural defense [5]. In addition to these physical protections, buds are often chemically defended: many plant species accumulate diverse suites of secondary metabolites within bud tissues, including compounds known to inhibit microbial growth and deter herbivory [2,6]. These chemical defenses are typically deployed as complex mixtures rather than as single compounds, yet the functional roles of many bud-associated metabolites remain poorly understood, as does the extent to which bud phytochemistry varies within and among species [7,8,9]. Characterizing bud phytochemistry across a diverse population provides a framework for understanding how chemical defenses vary within and among species and contribute to protection against biotic and abiotic stressors.

Among the plants with buds highly enriched in secondary metabolites are species of the genus *Populus* [10,11]. Poplar buds are chemically complex tissues containing abundant secondary metabolites that can be broadly grouped into major chemical classes, including phenylpropanoids, terpenoids, and linear hydrocarbons [8]. In some species, these compounds accumulate in resinous bud exudates that coat developing tissues [10,11]. Because these metabolites exhibit a wide range of physicochemical properties, comprehensive characterization of poplar bud chemistry requires multiple analytical approaches, and much of the chemical space in poplar buds remains insufficiently explored [10,12]. Most species-level surveys rely on a single accession as representative, providing only a narrow view of phytochemical diversity [8]. The true breadth of chemical diversity of bud secondary metabolites among members of natural *Populus* populations is largely undetermined.

Variation in bud phytochemistry can occur along multiple interpretable axes, including differences in the relative abundance of major chemical classes, the diversity of compounds within those classes, and the dominance of particular biosynthetic pathways. In *Populus* buds, phenylpropanoids and terpenoids frequently represent the most abundant and functionally significant metabolite groups [13,14]. Terpenoids, in particular, encompass structurally diverse mono-, sesqui-, and oxygenated compounds that have been widely implicated in chemical defense across plant taxa [15]. Phenylpropanoids, in particular flavonoids, flavonoid chalcones, and hydroxycinnamic acids and esters have been previously reported as major constituents of poplar bud exudates, and many of these compounds are implicated in bioactivity [13,14,16]. Linear hydrocarbons have also been described, and are generally known to be components of protective resins and cuticular waxes [13,14,17]. Despite the putative biological importance of these classes, the extent to which variation in their composition and relative concentration contributes to chemical defense remains poorly resolved, especially within species.

This gap is evident for *Populus trichocarpa*, one of the most extensively studied model species within the genus and a foundational system for understanding woody plant biology [18,19,20]. Native to western North America, *P. trichocarpa* occupies a broad geographic range encompassing diverse climatic and ecological conditions, from coastal temperate regions to continental and subalpine environments [21,22,23]. This range is reflected in a large, genetically diverse population comprising approximately 1000 distinct genotypes collected from British Columbia (Canada) to Lake Tahoe, providing a powerful framework for examining intraspecific variation in adaptive traits [24,25]. Population-level studies in *P. trichocarpa* have revealed extensive natural variation across a wide array of physiological and molecular processes, underscoring the species’ utility for linking genetic diversity to functional outcomes [24,25].

In this study, we leveraged this *P. trichocarpa* population to address a fundamental gap in our understanding of bud phytochemistry by profiling metabolite composition across a representative subset of the genotypes. Specifically, we aimed to determine the extent and patterns of natural variation in bud secondary metabolite composition and identify chemotypic groups within the species. Given that *Populus* secondary metabolites are known to serve diverse biological functions [3,26], we also used antifungal bioassays to explore the ecological relevance of this chemical variation by associating differences in the antifungal activity of bud extracts with differences in their underlying phytochemical composition. *Fusarium oxysporum*, a soilborne fungus representing a significant agricultural and ecological challenge due to its broad host range, persistence in soil, and increasing resistance to conventional fungicides [27,28], was chosen as our model fungal species for the bioassays.

## 2. Results and Discussion

### 2.1. Buds of the P. trichocarpa Reference Genotype Nisqually-1 Contain Complex Lipophilic Phytochemicals

The reference genome of *P. trichocarpa* was generated from the Nisqually-1 genotype, which has been utilized extensively as a model woody plant [29]. Accordingly, our initial chemical profiling of dormant bud tissues was conducted using Nisqually-1. Bud phytochemicals were extracted from ground fresh buds with ethyl acetate and analyzed by gas chromatography–mass spectrometry (GC–MS). Ethyl acetate was selected as the extraction solvent due to its intermediate polarity, enabling efficient recovery of both apolar terpenoids and moderately polar phenolic compounds while avoiding extensive co-extraction of highly polar primary metabolites that would complicate chromatographic separation [30,31,32]. Alternative solvents such as methanol extract broader compound ranges but include excessive polar interferents, while hexane and dichloromethane selectively extract only the most lipophilic compounds, potentially excluding important semi-polar defensive metabolites [32]. Because ethyl acetate preferentially extracts lipophilic and moderately apolar metabolites and GC–MS is best suited to volatile and semi-volatile compounds, subsequent analyses focus explicitly on lipophilic phytochemicals detected by this method [28]. LC–MS (liquid chromatography–mass spectrometry) is another widely used platform for chemical profiling and has previously been used to characterize poplar bud metabolites [33]. However, it is primarily employed for detection of polar compounds, which were not the chemicals of interest in this study. Subsequent analysis of buds by LC-MS would provide more comprehensive coverage of their array of phytochemicals. GC–MS is generally preferred for analysis of volatile compounds—such as mono- and sesqui-terpenoids—which are the primary target of this study [34]. Although many compounds found in dormant buds exhibit limited volatility, such as phenylpropanoids, certain derivatives may nevertheless be detected under our GC–MS conditions [35]. Future studies would also benefit from complementary analytical approaches such as GC-MS with derivatization to improve characterization of nonvolatile compounds, and HPLC coupled with diode array detection (HPLC-DAD) or UHPLC-based methods to improve characterization of polar and semi-polar metabolites that may be underrepresented in our analyses [36,37].

Using our GC-MS-based approach, a complex mixture of lipophilic phytochemicals was identified (Figure S1), amounting to a total of 32 metabolites in this line alone (Table 1). Detected compounds were classified into three major lipophilic chemical classes: phenylpropanoids (16 compounds), terpenoids (11 compounds), and linear hydrocarbons (4 compounds). A small number of additional detected features, representing chemically heterogeneous compounds outside these major classes, together contributed a substantial minority of the total chromatographic signal and were grouped separately for subsequent analyses.

Relative quantification using an internal standard showed that phenylpropanoids were the most abundant lipophilic chemical class in the Nisqually-1 bud extract, accounting for approximately 49% of the total chromatogram peak area, followed by terpenoids (~25%). Linear hydrocarbons contributed approximately 6% of the total chromatogram peak area. The remaining signal, accounting for roughly one-fifth of the chromatogram, was attributable primarily to a small number of compounds outside the three major classes, including sugars and sulfoxides. Together, these results indicate that dormant bud chemistry in the reference genotype is dominated by aromatic and terpenoid metabolites, while also containing a chemically heterogeneous background that contributes appreciably to overall lipophilicity.

The phenylpropanoid class was chemically diverse, comprising flavonoid, benzenoid, and other phenolic compounds. Benzyl 2-methoxybenzoate was the most abundant phenylpropanoid and the most abundant lipophilic compound overall, followed by benzyl salicylate and piceol. Additional phenylpropanoids included several benzoic-acid derivatives. Eleven terpenoids were identified, including monoterpene alcohol derivatives and abundant sesquiterpene alcohols such as cryptomeridiol and (–)-10-epi-γ-eudesmol. Linear hydrocarbons were represented primarily by straight-chain alkanes of varying length, including eicosane and nonacosane. These compounds likely reflect surface-associated or structural lipid components of dormant buds rather than specialized secondary metabolites [17]. Taken together, these data establish a chemically rich but structured lipophilic profile for the *P. trichocarpa* reference genotype and provide a baseline for evaluating population-level variation.

### 2.2. Variation in Bud Phytochemistry Among a P. trichocarpa Population

**Figure 1:**
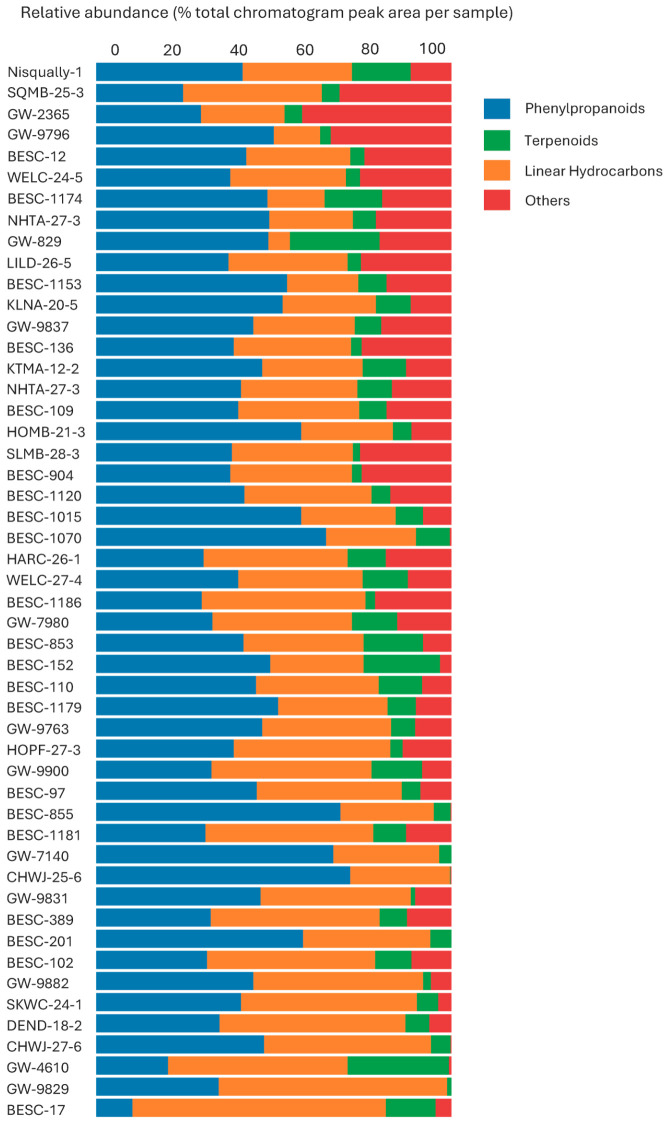
Chemical class composition of dormant bud extracts among *P. trichocarpa* genotypes. Stacked bar chart depicting relative class abundance, expressed as percent total chromatogram peak area within each sample (i.e., class abundances normalized to a per-sample total of 100%), across 50 *P. trichocarpa* genotypes. Chemical classes are indicated by color: phenylpropanoids (blue), terpenoids (green), linear hydrocarbons (orange), and others (red).

To determine whether lipophilic bud phytochemical profiles vary among members of the wild *P. trichocarpa* population, we analyzed dormant buds from 49 lines collected across the western United States and grown at a common garden at the University of California, Davis [24,25]. Buds were processed as for Nisqually-1, and detected metabolites were classified into phenylpropanoids, terpenoids, and linear hydrocarbons to enable direct comparison. Across the population panel, chromatographic profiles contained hundreds of detectable peaks representing diverse lipophilic metabolites (Table S1). The number of detected metabolites per genotype ranged from 11 to 80 (Table 2). Phenylpropanoids were the dominant chemical class across the panel, accounting on average for approximately 51% of the normalized chromatographic signal, followed by terpenoids (~13%) and linear hydrocarbons (~13%) (Figure 1). A chemically heterogeneous group of additional metabolites, including amides, ketones, and related compounds outside the major lipophilic classes, contributed the remaining ~22% of the signal. Most genotypes contained representatives of all major classes, although their relative abundance and chemical complexity varied substantially among lines.

Despite this extensive quantitative variation, a small subset of metabolites was detected at high frequency across the population. Benzyl 2-methoxybenzoate occurred in all genotypes examined and, excluding an outlier in GW-10072 which exhibited a particularly high concentration of this compound, had a mean abundance of 58.44 µg of 1-octanol equivalent per gram fresh weight. Benzyl benzoate and 4′-hydroxyacetophenone were also common, being detected in more than half of the lines within the dataset. These widespread and abundant phenylpropanoids define a conserved chemical core of dormant bud chemistry, suggesting that key biosynthetic activities are maintained across the population.

Beyond this conserved core, most detected metabolites occurred less frequently and exhibited high variability in abundance. These compounds spanned all three major lipophilic classes and accounted for much of the observed chemical diversity. This pattern indicates that population-level variation in bud chemistry is layered upon a shared biochemical foundation, with diversity arising through differences in relative abundance and compound occurrence rather than wholesale replacement of dominant metabolites.

### 2.3. Diverse P. trichocarpa Lines Cluster into Distinct Chemotypic Classes

**Figure 2:**
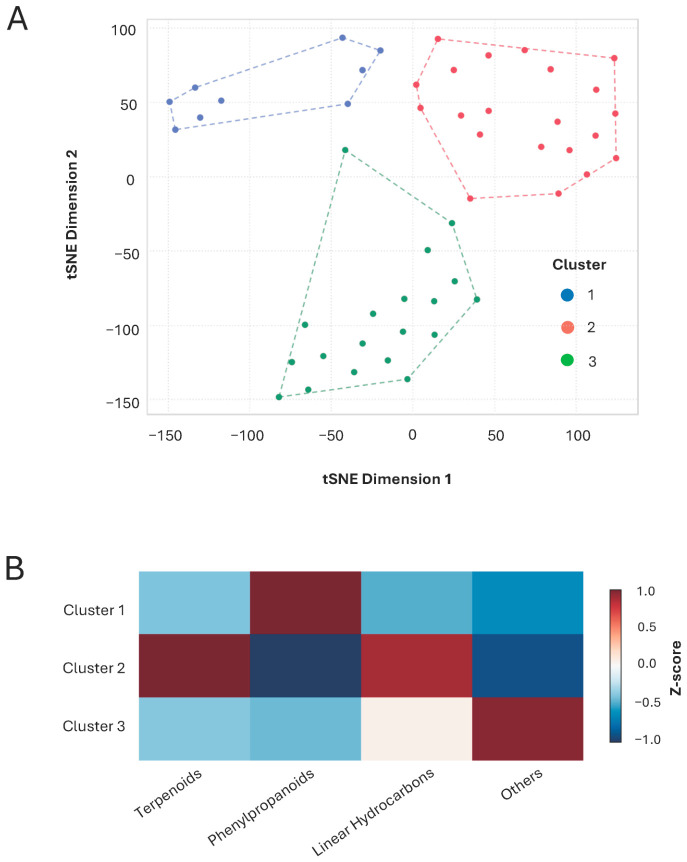
Population-level variation in bud phytochemistry across *P. trichocarpa* natural variants. (**A**) t-SNE ordination of Hellinger-transformed GC–MS profiles from ethyl acetate bud extracts, with k-means partitioning (k = 3; perplexity = 30). Each point represents one genotype; point size reflects total metabolite abundance. Shaded convex hulls delineate the three phytochemical clusters (red, green, blue). (**B**) Heatmap of Z-score–normalized relative abundance of major metabolite classes across the three clusters derived from genotype-level bud phytochemical profiles.

To determine whether population-level chemical variation is structured, we applied t-distributed stochastic neighbor embedding (t-SNE), a nonlinear dimensionality-reduction method that represents samples in a low-dimensional space by preserving local similarity relationships among high-dimensional observations [38]. This approach is well suited to complex metabolomic datasets for its emphasis on neighborhood structure rather than global variance, allowing chemically similar samples to group together even when relatively minor differences are distributed across a large set of compounds [39]. We performed t-SNE on Hellinger-transformed GC–MS abundance data, followed by k-means clustering (k = 3) [40,41]. This analysis resolved three discrete chemotypic clusters within the population panel (Figure 2A). The clusters differed primarily in the relative abundance of the major lipophilic chemical classes identified in dormant buds (Figure 2B). Cluster 1 was strongly enriched in phenylpropanoids relative to the remainder of the population, while exhibiting comparatively reduced contributions from terpenoids, linear hydrocarbons, and chemically heterogeneous “other” compounds. In contrast, Cluster 2 displayed elevated relative abundance of both terpenoids and linear hydrocarbons, coupled with markedly reduced phenylpropanoid representation. Cluster 3 was distinguished primarily by strong enrichment of the “other” compound category, with comparatively intermediate hydrocarbon abundance and below-average representation of both terpenoids and phenylpropanoids. Importantly, all clusters retained representatives of each major lipophilic class, indicating that chemotypic differentiation within the population reflects quantitative redistribution among broadly shared classes of metabolites rather than complete gain or loss of particular biosynthetic pathways. Together, these results demonstrate that dormant bud phytochemistry in *P. trichocarpa* is structured into discrete population-level chemotypes despite substantial overlap in overall qualitative composition.

### 2.4. Dormant Bud Extracts Significantly Inhibit Fungal Growth

**Figure 3:**
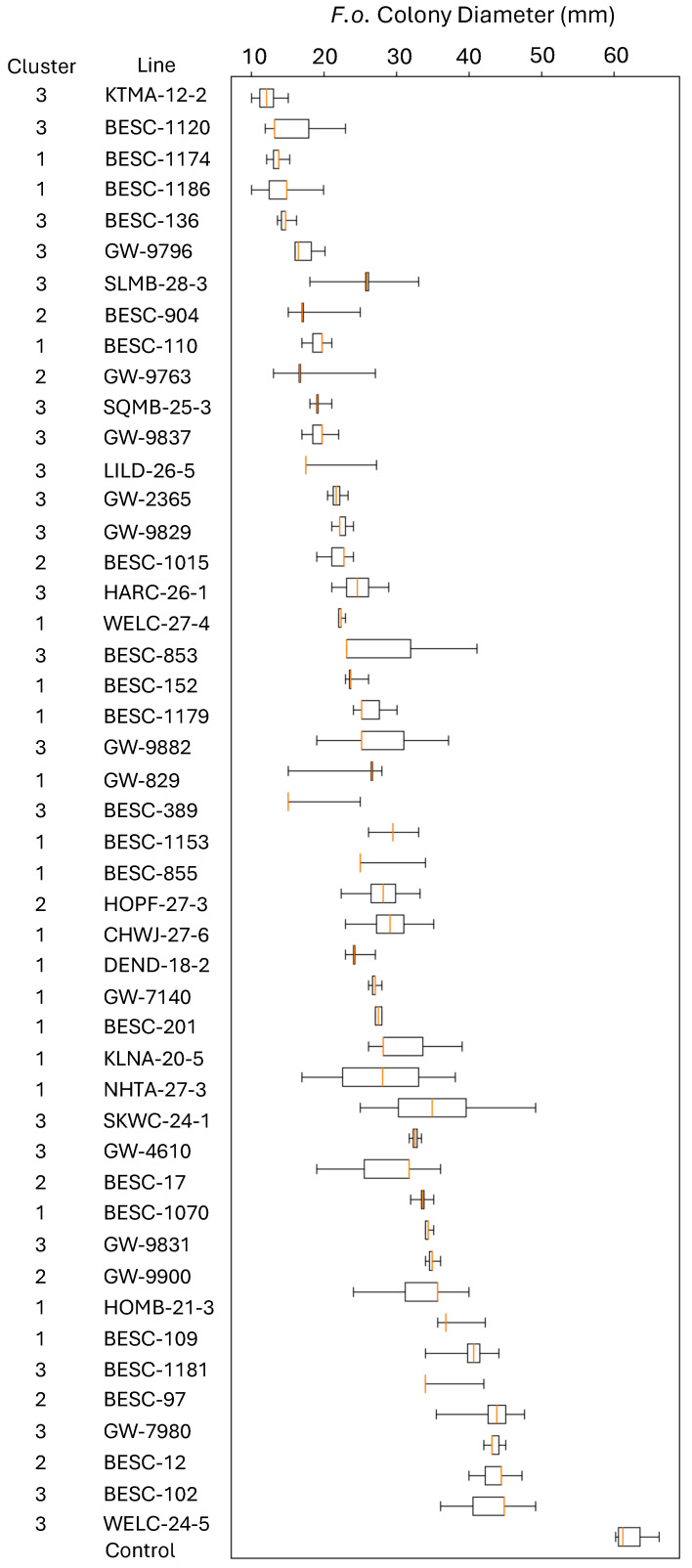
Bud extracts of *P. trichocarpa* inhibit *F. oxysporum* growth across all lines but vary in inhibition strength. Boxplots show the distribution of fungal colony diameter for each of the 50 *P. trichocarpa* bud extracts tested. The outlier at the far right represents the mock control. One-way ANOVA revealed significant differences in inhibition among lines (F_46,76_ = 5.53, *p* < 0.001).

**Figure 4:**
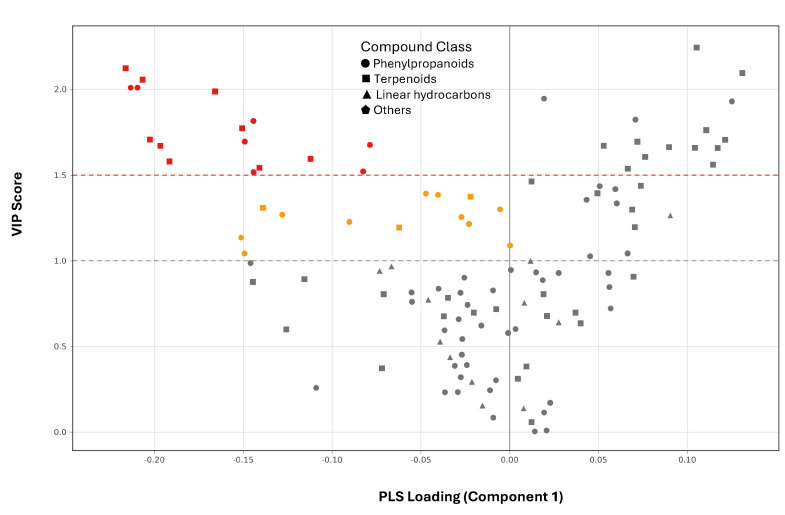
Identification of metabolites associated with antifungal activity. Variable Importance in Projection (VIP) scores were derived from partial least squares (PLS) regression of *P. trichocarpa* bud metabolite abundance against *F. oxysporum* colony diameter. Each point represents one compound, positioned by its PLS loading (*x*-axis) and VIP score (*y*-axis). Tier 1 (VIP > 1.5) and Tier 2 (VIP 1.0–1.5) antifungal candidates are shown in red and orange, respectively; marker shapes indicate compound class.

To assess whether variation in bud phytochemistry is associated with antifungal activity, ethyl acetate extracts from buds of 50 *P. trichocarpa* genotypes were applied to agar inoculated with the generalist fungal pathogen *Fusarium oxysporum* [27]. All extracts significantly inhibited fungal growth relative to solvent controls (Figure 3). Inhibition values formed a continuous distribution, and although line identity significantly affected mean colony diameter, no extract produced extreme inhibition relative to the remainder of the panel. To identify chemical features associated with variation in fungal growth inhibition, GC–MS profiles were integrated with colony diameter measurements using partial least squares regression [42]. One latent component captured the majority of covariance between chemical composition and fungal growth inhibition (R^2^ = 0.47). Variable importance analysis revealed 37 antifungal metabolites with VIP scores exceeding 1.0, distributed primarily across sesquiterpenes and phenylpropanoids rather than concentrated in a single compound class (Figure 4, Table 3) [43,44]. Among high-priority antifungal candidates (VIP > 1.5), sesquiterpenes and phenylpropanoids comprised 30 of the 37 total high VIP compounds across all chemical classes, with 21 compounds exhibiting antifungal activity as exhibited by negative PLS loadings.

Terpenoids demonstrated the highest mean VIP scores among antifungal compounds (mean VIP = 1.73), with α-farnesene and aromandendrene representing the top contributors. These compounds exhibited both high model importance and substantial effect magnitudes (PLS loadings ranging from −0.024 to −0.214) (Table 3). The terpenoid antifungal signature was dominated by sesquiterpenes (12 compounds), including multiple cadinene and murolene derivatives, with monoterpenes contributing four compounds primarily represented by linalool oxide variants (cis-pyranoid linalool oxide, 1,2-oxolinalool, and trans-linalool oxide). Phenylpropanoids showed strong representation among antifungal candidates (14 compounds, mean VIP = 1.35), led by (E)-hinokiresinol (VIP = 1.803) and included diverse benzoic acid derivatives, cinnamate esters, and hydroxylated aromatics (Table 3). The distribution of high VIP scores across multiple terpenoid and phenylpropanoid compounds (30 of 37 total antifungal compounds), rather than concentration within a narrow structural class, indicates that variation in antifungal activity among *P. trichocarpa* genotypes is associated with diverse chemical features spanning multiple compound classes. No single compound or tightly related chemical subgroup dominated the antifungal signature, indicating a broad-spectrum defensive chemistry profile involving both specialized sesquiterpene metabolism and general phenylpropanoid biosynthesis.

Notably, genotypes belonging to different chemotypic clusters frequently exhibited comparable inhibitory effects, despite clear differences in the proportional representation of major lipophilic chemical classes (Figure 3). Likewise, variable importance was distributed across numerous terpenoids and phenylpropanoids rather than concentrated on dominant individual predictors (Figure 4, Table 3). Together, these patterns argue against a model in which antifungal activity is determined largely by one highly bioactive metabolite or by membership in a particular chemotypic class. Instead, they suggest that inhibition reflects the combined contribution of multiple constituents whose relative abundances vary among genotypes, such that different chemical configurations yield highly similar antifungal outcomes. In this context, antifungal activity in dormant buds is best understood as an emergent property of the overall lipophilic mixture rather than as the effect of any single compound class, individual metabolite, or discrete chemical phenotype. Together, these results suggest that bud defense in *P. trichocarpa* is a compositional property of the chemical mixture, shaped by quantitative variation within a conserved metabolic repertoire.

## 3. Conclusions

This study provides a population-scale characterization of dormant bud phytochemistry in *P. trichocarpa*, revealing a chemically rich lipophilic metabolite landscape. Across all genotypes examined, bud extracts were dominated by phenylpropanoids, terpenoids, and linear hydrocarbons, forming a conserved chemical core shared throughout the population. Superimposed on this shared foundation, substantial quantitative variation in metabolite abundance and chemical class investment gave rise to distinct chemotypic groupings. These results demonstrate that intraspecific variation in bud chemistry reflects coordinated modulation of common biosynthetic pathways rather than qualitative differences in compound presence or absence. Functional assays showed that bud extracts from all genotypes significantly inhibited fungal growth, indicating that chemical defense is a ubiquitous property of *P. trichocarpa* buds. In the PLSR-VIP analysis, compounds associated with reduced fungal colony diameter were spread across multiple metabolites spanning both phenylpropanoid and terpenoid classes, with importance scores distributed rather than concentrated in a few clearly dominant predictors. These results suggest that antifungal activity reflects broader differences in metabolite mixture composition among genotypes rather than dependence on a single chemotype or a small number of key compounds. This chemically diverse mode of defense, characterized by numerous related but variable phenylpropanoid and terpenoid compounds, may provide a robust population-wide strategy that limits vulnerability to pathogen adaptation. From an applied perspective, these findings imply that improving bud defense through breeding or genetic manipulation is more likely to involve selection on pathway-level regulation and allocation patterns than on individual biosynthetic enzymes. More broadly, this work highlights dormant buds as an important and underexplored site of chemical defense and provides a framework for linking natural variation in secondary metabolism to functional outcomes in long-lived woody plants.

## 4. Materials and Methods

### 4.1. Plant Materials and Sample Collection

Poplar bud material was collected from a 6.1-ha plot at the Plant Science Field Facility of University of California at Davis (38°32′47.4″ N, 121°47′32.7″ W), as described previously [24]. The *P. trichocarpa* Torr. & Gray population used in this study was sourced from a range of latitudes exhibiting diverse climate and rainfall patterns, representative of most of the species’ habitats (38.9–54.3° N, 116–128.7° W), and included a total of 1382 genotypes. *P. trichocarpa* shoots bearing dormant buds were collected in January 2022 and stored at −80 °C until preparation of bud extracts.

### 4.2. GC-MS Analysis of Metabolites in Ethyl Acetate Extracts

Frozen buds were manually removed from shoots and ground with a mortar and pestle in liquid nitrogen. Since the collected branches bore both terminal and axillary buds, the ground bud tissue mixture contained both types. Next, 0.33 g of fine powder was mixed with 5 mL of ethyl acetate (1:15) and rocked for 30 min at 24 °C. The extracts were transferred to 2 mL tubes and centrifuged at 13,000 rpm, 4 °C, for 10 min. Supernatant was transferred to new tubes. Extracts filtered using a sterile syringe and Agilent 0.2 µm PTFE filters and were diluted 1:10 with ethyl acetate containing 0.0005% nonyl acetate as an internal standard. Extracts were stored at −80 °C until GC-MS analysis. Bud extracts were analyzed using a Shimadzu 17A gas chromatograph coupled to a Shimadzu QP5050A quadrupole mass selective detector. Compound separation was performed on a Restek SHR5XLB capillary column (30 m × 0.25 mm internal diameter × 0.25 µm film thickness; Shimadzu, Columbia, MD, USA). Helium was used as the carrier gas at a column flow rate of 0.96 mL min^−1^. Samples were injected at a volume of 1.5 µL using splitless injection mode with an injector temperature of 250 °C and a splitless sampling time of 1.5 min. The oven temperature program consisted of an initial temperature of 60 °C held for 6 min, followed by a ramp of 5 °C min^−1^ to 300 °C and a final hold at 300 °C for 5 min. Mass spectra were acquired using electron ionization (EI) at 70 eV over a scan range of *m*/*z* 43–350 with a solvent cut time of 1.5 min and an event time of 0.30 s. The ion source and interface temperatures were maintained at 200 °C and 280 °C, respectively. Products were identified based on the National Institute of Standards and Technology (NIST) mass spectral database by comparing retention times and mass spectra with authentic reference compounds where available. Compound quantification was performed using a total ion current (TIC) internal standard normalization method. Prior to extraction, nonyl acetate was added to each sample at a final concentration of 0.0005% (*w*/*v*) as an internal standard. Each sample consisted of 0.33 g of plant material extracted in 5 mL of solvent, yielding an internal standard quantity of 0.05 µL. The relative abundance of each compound was calculated as: relative abundance = (compound TIC peak area/IS TIC peak area) × (plant material mass [mg]/IS amount), where plant material mass was 330 mg per sample. Results represent relative abundance indices normalized to the internal standard and are not corrected for compound-specific ionization efficiency differences.

### 4.3. Fungal Growth Inhibition Assays

400 μL of centrifuged, undiluted ethyl acetate bud extracts were pipetted onto 90 mm petri plates of potato dextrose agar prepared according to the manufacturer’s directions. Extracts were spread with sterile plate spreaders and dried for approximately 30 min to allow for ethyl acetate evaporation. Control plates were spread with 400 μL of pure ethyl acetate. Three-day-old 9 mm^2^ mycelial plugs of *F. oxysporum* sp. *Lycopersici*, originally isolated from the Organic Crop Unit, University of Tennessee [45], were cut by sterile scalpel and placed in the center of the 90 mm petri dishes, mycelia side down. Plates were incubated at 28 °C for three days, and the diameters of the fungal colonies were measured at their widest point. Any plates with mycelial growth emanating from multiple points on the agar were discarded.

### 4.4. Clustering Analysis

Unsupervised clustering analyses were performed using bud extract GC–MS data from 50 *P. trichocarpa* genotypes. For each genotype, peak areas were first normalized relative to the internal standard and then expressed as relative abundances within samples. The data were then Hellinger transformed prior to multivariate analysis. Dimensionality reduction was performed using t-distributed stochastic neighbor embedding (t-SNE), a non-linear ordination method well suited for high-dimensional metabolomic datasets. The transformed data were embedded into two dimensions using a perplexity parameter of 30 and a fixed random seed. The resulting low-dimensional representation was used as input for k-means clustering. Based on inspection of within-cluster variance and cluster separation, the number of clusters was set to k = 3. Cluster assignments were used solely to describe patterns of chemical similarity and were not assumed to represent discrete biological categories. To characterize chemical differences among clusters, lipophilic metabolites were grouped into three major chemical classes as described above, and class-level abundances were averaged across genotypes within each cluster. Enrichment or depletion of chemical classes was visualized using z-score-scaled class means relative to the population average.

### 4.5. PLSR-VIP Analysis

Partial least squares regression (PLSR) was used to integrate metabolite abundance data with fungal growth inhibition measurements. Prior to analysis, 63 contaminant compounds and analytical artifacts were systematically removed from the chemical dataset based on expert curation. Chemical abundance data from 49 *P. trichocarpa* genotypes with overlapping chemical and fungal response measurements were standardized using Z-score normalization and analyzed against colony diameter measurements as the response variable. PLSR models were fitted using Python (version 3.14.5) scikit-learn, with the optimal number of latent components determined by minimizing prediction error during 5-fold cross-validation. Cross-validation confirmed that one component provided optimal predictive performance (R^2^ = 0.47), with additional components contributing primarily noise. Variable Importance in Projection (VIP) scores were calculated using the standard formula that weights each variable’s contribution to the latent components by the proportion of Y-variance explained by each component. VIP scores reflect each metabolite’s relative importance for the PLS model relating chemical composition to antifungal activity. Metabolites with VIP scores exceeding 1.5 were classified as highly important contributors, while those between 1.0 and 1.5 were considered moderately important, following established chemometrics conventions. Antifungal candidates were identified as compounds with negative PLS loadings (indicating decreased abundance with larger colony diameters) and VIP scores above 1.0. PLSR results were used to identify chemical features statistically associated with variation in antifungal activity and were not interpreted as evidence of direct causal effects.

## Figures and Tables

**Table 1:** Chemical profile of dormant buds of *P. trichocarpa* ‘Nisqually-1’.

Class	Subclass	Name	Percent Composition
phenylpropanoids	phenol	4-Vinylphenol	1.39
	acetophenone	Piceol	6.38
	benzenoid	Benzene, 1,1′-(1,2-cyclobutanediyl)bis-, trans-	3.58
	benzenoid	Benzyl Benzoate	3.62
	benzenoid	Benzoic acid, hept-2-yl ester	0.94
	benzenoid	Benzyl salicylate	6.44
	benzenoid	Benzyl 2-methoxybenzoate	14.85
	flavonoid	Pinostrobin	2.39
	benzenoid	Cyclohexane, 1,3,5-triphenyl-	5.60
	flavonoid	2′,6′-Dihydroxy 4′-methoxydihydrochalcone, diacetate	8.86
	benzenoid	Benzenepropanoic acid, 3-phenyl-2-propenyl ester	1.12
	flavonoid	2′,6′-Dihydroxy-4′-methoxychalcone	3.00
	flavonoid	5-Hydroxy-4′,7-dimethoxyflavanone	1.86
terpenoids	monoterpene	Eucalyptol	1.20
	monoterpene	2-hydroxy-1,8-cineol	6.58
	monoterpene	4-terpineol	0.90
	monoterpene	(-)-alpha-fenchol	2.59
	sesquiterpene	Nerolidol	0.27
	sesquiterpene	β-Eudesmol	3.38
	sesquiterpene	7-(2-Hydroxypropan-2-yl)-1,4a-dimethyldecahydronaphthalen-1-ol	1.01
	sesquiterpene	Cryptomeridiol	6.54
	sesquiterpene	2-Naphthalenemethanol, decahydro-alpha,alpha,4a-trimethyl-8-methylene-, [2R-(2 alpha,4a alpha, 8a beta)]-	4.75
	sesquiterpene	(−)-10-epi gamma-Eudsemol	5.14
	sesquiterpene	Hinesol	0.27
linear hydrocarbons	alkane	Tetradecane	0.85
	alkane	Eicosane	0.41
	alkane	Tetracosane	0.57
	alkane	Nonacosane	2.77
other	ketone	2,4-Pentanedione, 3-(1-methyl-2-propenyl)-	0.48
	pyrroline	2-Acetyl-1-pyrroline	0.76
	ketone	2-(2,2-Dimethylpropanoyl)cyclohexanone	0.33
	amide	9-Octadecenamide	1.20

**Table 2:** Diversity of bud phytochemistry by chemical class across *P. trichocarpa* genotypes.

Genotype	Phenylpropanoid	Terpenoid	Linear Hydrocarbon	Other
BESC-103	10	7	5	3
BESC-1164	12	9	3	6
BESC-1185	27	28	6	12
BESC-1183	15	3	10	8
BESC-129	5	8	8	6
BESC-162	9	3	6	4
BESC-163	11	4	5	2
BESC-164	9	1	6	3
BESC-165	13	2	9	5
BESC-261	23	18	7	15
BESC-262	12	4	6	28
BESC-29	8	5	15	15
BESC-290	15	4	13	8
BESC-291	18	20	5	4
BESC-292	13	2	9	5
BESC-293	12	9	3	6
BESC-368	17	11	5	6
BESC-485	32	28	6	12
BESC-6	17	14	9	16
BESC-904	9	5	15	14
DENA-17-1	20	6	9	2
DENA-17-2	9	1	6	3
DENA-17-3	12	4	8	6
DENA-17-4	13	3	9	6
DENB-17-1	27	25	7	15
GW-10072	5	1	2	3
GW-10073	17	4	5	11
GW-10989	27	25	7	15
GW-4580	6	5	7	6
GW-4583	5	8	8	6
GW-4584	11	2	6	3
GW-4585	7	17	6	3
GW-4588	9	3	6	3
GW-7091	10	7	5	3
GW-7094	5	1	2	3
GW-7096	4	2	6	7
GW-7098	27	28	6	12
GW-7109	17	4	5	11
GW-7647	6	5	7	6
GW-7648	7	4	6	4
GW-8489	15	4	13	8
GW-9816	11	2	6	3
GW-9817	13	3	9	6
GW-9903	12	4	8	6
GW-9947	18	20	5	4
GW-9964	12	3	8	5
LILB-26-3	21	1	6	4
NHTA-27-5	10	6	8	6
WELC-27-3	17	14	9	5
Nisqually-1	12	11	4	4

**Table 3:** Top-ranked metabolites associated with fungal growth inhibition.

Rank	Compound Name	VIP ^a^ Score	PLS ^b^ Loading	Chemical Class	Tier
1	Alpha-farnesene	2.107	−0.214	Terpenoids (Ses. ^c^)	Tier 1 (High VIP)
2	Aromandendrene	2.043	−0.203	Terpenoids (Ses.)	
3	Palmitoylethanolamide	2.013	−0.210	Others	
4	Zonarene	2.011	−0.2101	Terpenoids (Ses.)	
5	1-epi-Cubenol	2.003	−0.2104	Terpenoids (Ses.)	
6	α-Cadinene	2.001	−0.2105	Terpenoids (Ses.)	
7	Copaene	1.997	−0.2106	Terpenoids (Ses.)	
8	α-Cadinol	1.983	−0.2115	Terpenoids (Ses.)	
9	α-Amorphene	1.947	−0.1607	Terpenoids (Ses.)	
10	(*E*)-hinokiresinol	1.803	−0.1463	Phenylpropanoids	
11	(*Z*)-linalool oxide (pyranoid)	1.760	−0.1468	Terpenoids (Mono.)	
12	α-Curcumene	1.760	−0.1468	Terpenoids (Ses.)	
13	tau-Cadinol	1.696	−0.2000	Terpenoids (Ses.)	
14	δ-Cadinene	1.682	−0.1451	Terpenoids (Ses.)	
15	2,2,5-trimethyl-5-(3-methylbut-2-enyl)-8-oxidanyl-7-(3-phenylpropanoyl)chromen-6-one	1.663	−0.0795	Phenylpropanoids	
16	γ-Cadinene	1.659	−0.1950	Terpenoids (Ses.)	
17	1,2-Oxolinalool	1.580	−0.1121	Terpenoids (Mono.)	
18	γ-Muurolene	1.568	−0.1896	Terpenoids (Ses.)	
19	Muurolol	1.531	−0.1374	Terpenoids (Ses.)	
20	3′-Hydroxyacetophenone	1.510	−0.0852	Others	
21	(E,E)-Methyl 10,11-epoxyfarnesoate	1.506	−0.1404	Terpenoids (Ses.)	
22	1,4-Diphenyl-1-butanone	1.420	−0.0513	Phenylpropanoids	Tier 2 (Medium VIP)
23	1-Chlorooctadecane	1.384	−0.0437	Linear Hydrocarbons	
24	(*Z*)-linalool oxide	1.382	−0.0516	Terpenoids (Mono.)	
25	N,N-Dimethylpalmitamide	1.382	−0.0516	Others	
26	(2E, 6E)-Farnesyl benzoate	1.382	−0.0516	Phenylpropanoids	
27	(−)-α-fenchol	1.366	−0.0242	Terpenoids (Mono.)	
28	Phenylethyl salicylate	1.299	−0.1358	Phenylpropanoids	
29	Pinostrobin Chalcone	1.290	−0.0034	Phenylpropanoids	
30	Sakuranetin	1.261	−0.1243	Phenylpropanoids	
31	2-Cyclohexyl-hex-5-en-2-ol	1.246	−0.0247	Others	
32	1-Ethyl-4-methoxybenzene	1.246	−0.0247	Phenylpropanoids	
33	Pinostrobin	1.218	−0.0891	Phenylpropanoids	
34	trans-Cinnamic acid	1.207	−0.0247	Phenylpropanoids	
35	Benzyl trans-4-coumarate	1.206	−0.0862	Phenylpropanoids	
36	2-Acetylphenol	1.186	−0.0577	Phenylpropanoids	
37	2,4-di-tert-butylphenol	1.127	−0.1506	Phenylpropanoids	

^a^ VIP: Variable Importance in Projection; ^b^ PLS: partial least squares regression. ^c^ “Ses.” and “Mono.” in parentheses stand for sesquiterpene and monoterpene, respectively.

## Data Availability

The authors declare that all data supporting the findings of this study are available within the paper and its Supplementary Information Files.

## Supplementary Materials

The following supporting information can be downloaded at: https://www.mdpi.com/article/10.3390/plants15111746/s1, Figure S1: GC chromatogram of organic extract of dormant buds of the Nisqually-1 genotype of *P. trichocarpa*; Table S1: Chemical Profiles of Dormant Bud Extracts from 49 *P. trichocarpa* Genotypes.
