# Supplementary material for: Phytochemical Diversity in Populus trichocarpa Buds: Insights into Population Variation and Antifungal Properties
Source: Plants (Basel). 2026 Jun 4;15(11):1746. doi: 10.3390/plants15111746 (PMC13258884; doi:10.3390/plants15111746)
Supplement: Supplementary file 1 [file plants-15-01746-s001.zip › plants-4289713-supplementary.pdf]

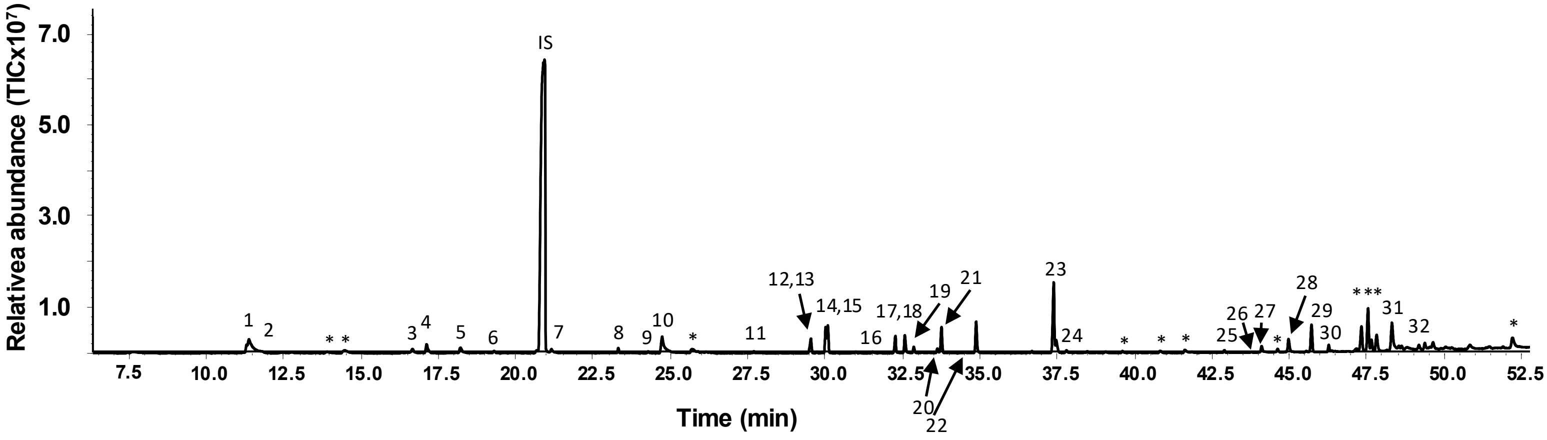

Figure S1. GC chromatogram of organic extract of dormant buds of the Nisqually-1 genotype of *P. trichocarpa*. 1. Eucalyptol; 2. 2-hydroxy-1,8-cineol; 3. 4-terpineol; 4. (-)-alpha-fenchol; 5. 4-vinylphenol; 6. 2,4-Pentanedione, 3-(1-methyl-2-propenyl)-; 7. 2-Acetyl-1-pyrroline; 8. Tetradecane; 9. 2-(2,2-Dimethylpropanoyl)cyclohexanone ;10. Piceol; 11. Nerolidol; 12. 7-(2-Hydroxypropan-2-yl)-1,4a-dimethyldecahydronaphthalen-1-ol; 13. Hinesol; 14. 2-Naphthalenemethanol, decahydro-alpha, alpha,4a-trimethyl-8-methylene-, [2R-(2 alpha,4a alpha,8a beta)]-; 15. (-)-10-epigamma-Eudsemol; 16. Benzene, 1,1'-(1,2-cyclobutanediyl)bis-, trans-; 17. Cyclohexane, 1,3,5-triphenyl-; 18. Benzyl Benzoate; 19. Cryptomeridiol; 20. Benzoic acid, hept-2-yl ester; 21. β-Eudesmol; 22. Benzyl salicylate; 23. Benzyl 2-methoxybenzoate ; 24. Eicosane; 25. 9-Octadecenamide; 26. Tetracosane; 27. Pinostrobin; 28. Pinostrobin chalcone; 29. Nonacosane; 30. 2',6'-Dihydroxy 4'-methoxydihydrochalcone, diacetate; 31. Benzenepropanoic acid, 3-phenyl-2-propenyl ester; 32. 5-Hydroxy-4',7-dimethoxyflavanone. Peaks denoted with an asterisk represent contaminant compounds. "IS" stands for the internal standard, nonyl acetate.

Table S1. Chemical Profiles of Dormant Bud Extracts from 49 *P. trichocarpa* Genotypes

Genotype	Class	Name
GW-829	Terpenoid	Eucalyptol
	Terpenoid	2-Oxabicyclo[2.2.2]octane, 1,3,3-trimethyl-
	Terpenoid	4-terpineol
	Terpenoid	(-)-alpha-fenchol
	Phenylpropanoids	4-Vinylphenol
	Others	2,4-Pentanedione, 3-(1-methyl-2-propenyl)-
	Others	2-Acetyl-1-pyrroline
	Linear Hydrocarbons	Tetradecane
	Others	2-(2,2-Dimethylpropanoyl)cyclohexanone
	Phenylpropanoids	piceol
	Terpenoid	1,6,10-Dodecatrien-3-ol, 3,7,11-trimethyl-, [S-(Z)]-
	Terpenoid	2-Naphthalenemethanol, 1,2,3,4,4a,5,6,7-octahydro-.alpha.,.alpha.,4a,8-tetramethyl-, (2R-cis)-
	Terpenoid	Hinesol
	Terpenoid	2-Naphthalenemethanol, decahydro-.alpha.,.alpha.,4a-trimethyl-8-methylene-, [2R-(2.alpha.,4a.alpha.,8a.beta.)]-
	Terpenoid	(-)-10-epi.gamma.-Eudsemol
	Phenylpropanoids	Benzene, 1,1'-(1,2-cyclobutanediyl)bis-, trans-
	Phenylpropanoids	Benzyl Benzoate
	Terpenoid	cryptomeridiol
	Phenylpropanoids	Benzoic acid, hept-2-yl ester
	Terpenoid	(1R,4aR,7R,8aR)-7-(2-Hydroxypropan-2-yl)-1,4a-dimethyldecahydronaphthalen-1-ol
	Phenylpropanoids	Benzoic acid, 2-hydroxy-, phenylmethyl ester
	Phenylpropanoids	2-Methoxybenzoic acid, benzyl ester
	Phenylpropanoids	2-Methoxybenzyl benzoate
	Linear Hydrocarbons	Eicosane
	Others	9-Octadecenamide
	Linear Hydrocarbons	Tetracosane
	Phenylpropanoids	4H-1-Benzopyran-4-one, 2,3-dihydro-5-hydroxy-7-methoxy-2-phenyl-, (S)-
	Linear Hydrocarbons	Nonacosane
	Phenylpropanoids	2',6'-Dihydroxy 4'-methoxydihydrochalcone, diacetate
	Phenylpropanoids	Benzenepropanoic acid, 3-phenyl-2-propenyl ester
	Phenylpropanoids	5-Hydroxy-4',7-dimethoxyflavanone
GW-4588	Linear Hydrocarbons	Tetradecane
	Others	Acetophenone, 4'-hydroxy-
	Others	1-(3-tricyclo[2.2.1.0(2.6)]heptyl)-1-butanone
	Terpenoid	1H-Benzocyclohepten-7-ol, 2,3,4,4a,5,6,7,8-octahydro-1,1,4a,7-tetramethyl-, cis-
	Terpenoid	1,6,10-Dodecatrien-3-ol, 3,7,11-trimethyl-, [S-(Z)]-
	Phenylpropanoids	Benzene, 1,1'-(1,2-cyclobutanediyl)bis-, trans-
	Phenylpropanoids	Benzyl Benzoate
	Phenylpropanoids	Benzoic acid, 2-hydroxy-, phenylmethyl ester
	Phenylpropanoids	2-Methoxybenzoic acid, benzyl ester
	Phenylpropanoids	(Z)-Cinnamyl benzoate
	Phenylpropanoids	Benzeneacetic acid, methyl ester
	Linear Hydrocarbons	Nonacosane
	Linear Hydrocarbons	Tetracosane
	Phenylpropanoids	4H-1-Benzopyran-4-one, 2,3-dihydro-5-hydroxy-7-methoxy-2-phenyl-, (S)-
	Others	2',6'-Dihydroxy 4'-methoxydihydrochalcone, diacetate
	Phenylpropanoids	Benzenepropanoic acid, 3-phenyl-2-propenyl ester
	Linear Hydrocarbons	Tricosane
	Terpenoid	Naphthalene, 1,2,3,4-tetrahydro-1-phenyl-
GW-4580	Others	Butanoic acid, 2-methyl-, ethyl ester
	Phenylpropanoids	Benzene, 1,3-dimethyl-
	Others	D-Limonene
	Linear Hydrocarbons	Undecane
	Terpenoid	(1S)-1,3,3-trimethylnorbornan-2-ol
	Linear Hydrocarbons	Dodecane
	Others	Acetophenone, 4'-hydroxy-
	Phenylpropanoids	Ethanone, 1-(3-hydroxyphenyl)-
	Terpenoid	1H-Benzocyclohepten-7-ol, 2,3,4,4a,5,6,7,8-octahydro-1,1,4a,7-tetramethyl-, cis-
	Terpenoid	1,6,10-Dodecatrien-3-ol, 3,7,11-trimethyl-, [S-(Z)]-
	Terpenoid	Farnesene epoxide, E-
	Phenylpropanoids	Benzene, 1,1'-(1,2-cyclobutanediyl)bis-, trans-
	Phenylpropanoids	2-Methoxybenzoic acid, benzyl ester
	Phenylpropanoids	2-Methoxybenzyl benzoate
	Linear Hydrocarbons	Nonacosane
	Linear Hydrocarbons	Tetracosane
	Linear Hydrocarbons	Tricosane
	Terpenoid	Naphthalene, 1,2,3,4-tetrahydro-1-phenyl-
	Others	Butanoic acid, 2-methyl-, ethyl ester
	Phenylpropanoids	Benzene, 1,3-dimethyl-
	Others	Heptane, 2,2,4,6,6-pentamethyl-
	Terpenoid	.alpha.-Methyl-.alpha.-[4-methyl-3-pentenyl]oxiranemethanol
	Terpenoid	Linalool
	Terpenoid	cis-pyranoid linalool oxide
	Others	Benzaldehyde, 4-hydroxy-
	Terpenoid	Copaene
	Phenylpropanoids	trans-Cinnamic acid
	Others	Acetophenone, 4'-hydroxy-
	Terpenoid	1H-Cycloprop[e]azulene, decahydro-1,1,7-trimethyl-4-methylene-, (1aR,4aS,7R,7aR,7bS)-(-)-
	Terpenoid	.gamma.-Muurolene
	Terpenoid	1,2,4-Metheno-1H-indene, octahydro-1,7a-dimethyl-5-(1-methylethyl)-, [1S-(1.alpha.,2.alpha.,3a.beta.,4.alpha.,5.alpha.,7a.beta.
	Phenylpropanoids	Benzoic acid, 2-methoxy-
	Terpenoid	Naphthalene, decahydro-4a-methyl-1-methylene-7-(1-methylethenyl)-, [4aR-(4a.alpha.,7.alpha.,8a.beta.)]-
	Others	2-Oxazolamine, 4,5-dihydro-5-(phenoxymethyl)-N-[(phenylamino)carbonyl]-
	Others	1,6-Dimethyl-4-propan-2-yl-1,2,3,7,8,8a-hexahydronaphthalene
	Terpenoid	Naphthalene, 1,2,4a,5,6,8a-hexahydro-4,7-dimethyl-1-(1-methylethyl)-
	Terpenoid	.alpha.-Farnesene
	Terpenoid	Naphthalene, 1,2,3,4,4a,5,6,8a-octahydro-7-methyl-4-methylene-1-(1-methylethyl)-, (1.alpha.,4a.beta.,8a.alpha.)-

GW-7098	Terpenoid	Naphthalene, 1,2,3,5,6,8a-hexahydro-4,7-dimethyl-1-(1-methylethyl)-, (1S-cis)-
	Terpenoid	Zonarene
	Terpenoid	Naphthalene, 1,2,3,4,4a,7-hexahydro-1,6-dimethyl-4-(1-methylethyl)-
	Terpenoid	Naphthalene, 1,2,4a,5,6,8a-hexahydro-4,7-dimethyl-1-(1-methylethyl)-, [1S-(1.alpha.,4a.beta.,8a.alpha.)]-
	Terpenoid	4a(2H)-Naphthalenol, 1,3,4,5,6,8a-hexahydro-4,7-dimethyl-1-(1-methylethyl)-, (1S,4R,4aS,8aR)-
	Terpenoid	4a(2H)-Naphthalenol, 1,3,4,5,6,8a-hexahydro-4,7-dimethyl-1-(1-methylethyl)-, (1S,4S,4aS,8aR)-
	Terpenoid	.tau.-Cadinol
	Terpenoid	1-Naphthalenol, 1,2,3,4,4a,7,8,8a-octahydro-1,6-dimethyl-4-(1-methylethyl)-, [1R-(1.alpha.,4.beta.,4a.beta.,8a.beta.)]-
	Terpenoid	.alpha.-Cadinol
	Phenylpropanoids	Benzene, 1,1'-(1,2-cyclobutanediyl)bis-, trans-
	Phenylpropanoids	Benzyl Benzoate
	Phenylpropanoids	Benzoic acid, 1-phenylethyl ester
	Phenylpropanoids	Benzoic acid, hept-2-yl ester
	Terpenoid	Neophytadiene
	Others	(2E,6E,9E)-2,6,10-Trimethyl-2,6,9,11-dodecatetraenal
	Others	Hexadecanamide, N-(2-hydroxyethyl)-
	Phenylpropanoids	Benzoic acid, 2-hydroxy-, phenylmethyl ester
	Others	2,6-Nonadienoic acid, 9-(3,3-dimethyloxiranyl)-3,7-dimethyl-, methyl ester, (E,E)-
	Others	n-Hexadecanoic acid
	Phenylpropanoids	Phenylethyl salicylate
	Phenylpropanoids	2-Methoxybenzoic acid, benzyl ester
	Phenylpropanoids	2-Methoxybenzyl benzoate
	Phenylpropanoids	Benzene, [[(1-ethenyl-1,5-dimethyl-4-hexenyl)oxy]methyl]-
	Others	Acetic acid, 3-hydroxy-6-isopropenyl-4,8a-dimethyl-1,2,3,5,6,7,8,8a-octahydronaphthalen-2-yl ester
	Phenylpropanoids	(Z)-Cinnamyl benzoate
	Phenylpropanoids	2-Methoxybenzoic acid, 2-phenylethyl ester
	Phenylpropanoids	Benzenemethanol, 2-hydroxy-3,6-dimethyl-4-(phenylmethoxy)-
	Phenylpropanoids	1-Butanone, 1,4-diphenyl-
	Phenylpropanoids	4-(Benzyloxy)-2-nitroaniline
	Linear Hydrocarbons	Tetracosane
	Phenylpropanoids	4H-1-Benzopyran-4-one, 2,3-dihydro-5-hydroxy-7-methoxy-2-phenyl-, (S)-
	Phenylpropanoids	Cinnamyl cinnamate
	Linear Hydrocarbons	Nonacosane
	Phenylpropanoids	4H-1-Benzopyran-4-one, 5-hydroxy-7-methoxy-2-phenyl-
	Phenylpropanoids	2',6'-Dihydroxy 4'-methoxydihydrochalcone, diacetate
	Phenylpropanoids	Benzenepropanoic acid, 3-phenyl-2-propenyl ester
	Phenylpropanoids	5-Hydroxy-4',7-dimethoxyflavanone
	Others	Phenol, 4,4'-(3-ethenyl-1-propene-1,3-diyl)bis-, (E)-
	Terpenoid	Naphthalene, 1,2,3,4-tetrahydro-1-phenyl-
	Phenylpropanoids	Sakuranetin
	Phenylpropanoids	Isosakuranetin, diacetate
	Linear Hydrocarbons	2-Methylhexacosane
	Terpenoid	Squalene
BESC-1153	Others	Butanoic acid, 2-methyl-, ethyl ester
	Phenylpropanoids	Benzene, 1,3-dimethyl-
	Others	Heptane, 2,2,4,6,6-pentamethyl-
	Terpenoid	.alpha.-Methyl-.alpha.-[4-methyl-3-pentenyl]oxiranemethanol
	Terpenoid	Linalool
	Terpenoid	cis-linalool oxide
	Phenylpropanoids	Benzoic acid
	Others	4-Vinylphenol
	Phenylpropanoids	Benzoic acid, 1-methylpropyl ester
	Terpenoid	Bicyclogermacrene
	Terpenoid	Copaene
	Phenylpropanoids	trans-Cinnamic acid
	Phenylpropanoids	Ethanone, 1-(2-hydroxyphenyl)-
	Phenylpropanoids	Ethanone, 1-(3-hydroxyphenyl)-
	Terpenoid	1H-Cycloprop[e]azulene, decahydro-1,1,7-trimethyl-4-methylene-, (1aR,4aS,7R,7aR,7bS)-(-)-
	Terpenoid	1-Isopropyl-4,7-dimethyl-1,2,3,4,5,6-hexahydronaphthalene
	Terpenoid	.gamma.-Muurolene
	Terpenoid	(1R,4aS,8aR)-1-Isopropyl-4,7-dimethyl-1,2,4a,5,6,8a-hexahydronaphthalene
	Phenylpropanoids	Benzoic acid, 2-methoxy-
	Others	Isopropyl-1,5,9-trimethyl-15-oxabicyclo[10.2.1]pentadeca-5,9-dien-2-ol
	Terpenoid	.alpha.-Muurolene
	Terpenoid	.alpha.-Farnesene
	Others	Phenol, 2,4-bis(1,1-dimethylethyl)-
	Terpenoid	Naphthalene, 1,2,3,4,4a,5,6,8a-octahydro-7-methyl-4-methylene-1-(1-methylethyl)-, (1.alpha.,4a.beta.,8a.alpha.)-
	Terpenoid	.delta.-Cadinene
	Terpenoid	Zonarene
	Terpenoid	Naphthalene, 1,2,3,4,4a,7-hexahydro-1,6-dimethyl-4-(1-methylethyl)-
	Terpenoid	Naphthalene, 1,2,4a,5,6,8a-hexahydro-4,7-dimethyl-1-(1-methylethyl)-, [1S-(1.alpha.,4a.beta.,8a.alpha.)]-
	Terpenoid	4a(2H)-Naphthalenol, 1,3,4,5,6,8a-hexahydro-4,7-dimethyl-1-(1-methylethyl)-, (1S,4R,4aS,8aR)-
	Terpenoid	4a(2H)-Naphthalenol, 1,3,4,5,6,8a-hexahydro-4,7-dimethyl-1-(1-methylethyl)-, (1S,4S,4aS,8aR)-
	Terpenoid	.tau.-Cadinol
	Terpenoid	1-Naphthalenol, 1,2,3,4,4a,7,8,8a-octahydro-1,6-dimethyl-4-(1-methylethyl)-, [1R-(1.alpha.,4.beta.,4a.beta.,8a.beta.)]-
	Terpenoid	.alpha.-Cadinol
	Others	Phenol, 2,4-di-t-butyl-6-nitro-
	Phenylpropanoids	4-Pentenoic acid, 5-phenyl-
	Phenylpropanoids	Benzene, 1,1'-(1,2-cyclobutanediyl)bis-, trans-
	Phenylpropanoids	Benzyl Benzoate
	Phenylpropanoids	Benzoic acid, 1-phenylethyl ester
	Phenylpropanoids	Benzoic acid, hept-2-yl ester
	Terpenoid	(1R,4aR,7R,8aR)-7-(2-Hydroxypropan-2-yl)-1,4a-dimethyldecahydronaphthalen-1-ol
	Terpenoid	alpha-sinesal
	Others	Hexadecanamide, N-(2-hydroxyethyl)-
	Phenylpropanoids	Benzoic acid, 2-hydroxy-, phenylmethyl ester
	Others	n-Hexadecanoic acid
	Phenylpropanoids	Phenylethyl salicylate
	Phenylpropanoids	2-Methoxybenzoic acid, benzyl ester
	Phenylpropanoids	2-Methoxybenzyl benzoate

	Phenylpropanoids	(Z)-Cinnamyl benzoate
	Phenylpropanoids	2-Methoxybenzoic acid, 2-phenylethyl ester
	Phenylpropanoids	Benzoic acid, 2-hydroxy-4-[(6-hydroxy-3,4-dimethoxy-2-methylbenzoyl)oxy]-6-methyl-, 3-hydroxy-5-methyl-4-[(phenylmethoxy)carbon
	Phenylpropanoids	4-(Benzyloxy)-3-methoxybenzoic acid
	Linear Hydrocarbon	Octadecanoic acid
	Phenylpropanoids	1-Butanone, 1,4-diphenyl-
	Linear Hydrocarbon	Tetracosane
	Phenylpropanoids	4H-1-Benzopyran-4-one, 2,3-dihydro-5-hydroxy-7-methoxy-2-phenyl-, (S)-
	Phenylpropanoids	Cinnamyl cinnamate
	Linear Hydrocarbon	2-Methylhexacosane
	Linear Hydrocarbon	Hexatriacontane
	Phenylpropanoids	4H-1-Benzopyran-4-one, 5-hydroxy-7-methoxy-2-phenyl-
	Phenylpropanoids	2',6'-Dihydroxy 4'-methoxydihydrochalcone, diacetate
	Phenylpropanoids	Benzenepropanoic acid, 3-phenyl-2-propenyl ester
	Linear Hydrocarbon	Nonacosane
	Phenylpropanoids	5-Hydroxy-4',7-dimethoxyflavanone
	Phenylpropanoids	(E)-hinokiresinol
	Terpenoid	Naphthalene, 1,2,3,4-tetrahydro-1-phenyl-
	Phenylpropanoids	Galangin
	Phenylpropanoids	4H-1-Benzopyran-4-one, 2,3-dihydro-5-hydroxy-2-(4-hydroxyphenyl)-7-methoxy-, (S)-
	Phenylpropanoids	Isosakuranetin, diacetate
	Others	Hexanoic acid, heptadecyl ester
	Others	Eicosanoic acid, 2-(acetyloxy)-1-[(acetyloxy)methyl]ethyl ester
BESC-1181	Others	Butanoic acid, 3-methyl-, ethyl ester
	Phenylpropanoids	Benzene, 1,3-dimethyl-
	Others	Heptane, 2,2,4,6,6-pentamethyl-
	Terpenoid	.alpha.-Methyl-.alpha.-[4-methyl-3-pentenyl]oxiranemethanol
	Linear Hydrocarbon	Undecane
	Terpenoid	Linalool
	Terpenoid	Estragole
	Terpenoid	1-Cyclohexene-1-carboxaldehyde, 2,6,6-trimethyl-
	Linear Hydrocarbon	Tetradecane
	Phenylpropanoids	2-Propenoic acid, 3-phenyl-
	Others	Acetophenone, 4'-hydroxy-
	Phenylpropanoids	Benzoic acid, 2-methoxy-
	Others	2,4-Di-tert-butylphenol
	Terpenoid	2-(4a,8-Dimethyl-2,3,4,5,6,7-hexahydro-1H-naphthalen-2-yl)propan-2-ol
	Terpenoid	Agarospirol
	Terpenoid	2-Naphthalenemethanol, decahydro-.alpha.,.alpha.,4a-trimethyl-8-methylene-, [2R-(2.alpha.,4a.alpha.,8a.beta.)]-
	Terpenoid	2-Naphthalenemethanol, 1,2,3,4,4a,5,6,8a-octahydro-.alpha.,.alpha.,4a,8-tetramethyl-, [2R-(2.alpha.,4a.alpha.,8a.beta.)]-
	Others	Phenol, 2,4-di-t-butyl-6-nitro-
	Phenylpropanoids	Benzene, 1,1'-(1,2-cyclobutanediyl)bis-, trans-
	Phenylpropanoids	Benzyl Benzoate
	Others	Bromoacetic acid, pentadecyl ester
	Terpenoid	2-Cyclohexen-1-one, 2-methyl-5-(1-methylethenyl)-
	Terpenoid	(1R,4aR,7R,8aR)-7-(2-Hydroxypropan-2-yl)-1,4a-dimethyldecahydronaphthalen-1-ol
	Terpenoid	(2E,6E,9E)-2,6,10-Trimethyl-2,6,9,11-dodecatetraenal
	Phenylpropanoids	Benzoic acid, 2-hydroxy-, phenylmethyl ester
	Others	1-Hexadecanol
	Others	Isoheptadecanol
	Others	1-Nonadecene
	Others	n-Hexadecanoic acid
	Phenylpropanoids	2-Methoxybenzoic acid, benzyl ester
	Phenylpropanoids	2-Methoxybenzyl benzoate
	Phenylpropanoids	(E)-Cinnamyl benzoate
	Others	9,12-Octadecadienoic acid (Z,Z)-, methyl ester
	Others	Benzamide, N-(2-cyanocyclopent-1-enyl)-3,4-dimethoxy-
	Linear Hydrocarbon	Tetracosane
	Phenylpropanoids	(-)-Pinostrobin
	Phenylpropanoids	Cinnamyl cinnamate
	Phenylpropanoids	2',6'-Dihydroxy 4'-methoxydihydrochalcone, diacetate
	Phenylpropanoids	Benzenepropanoic acid, 3-phenyl-2-propenyl ester
	Linear Hydrocarbon	Nonacosane
	Others	5-Hydroxy-4',7-dimethoxyflavanone
	Others	Phenol, 4,4'-(3-ethenyl-1-propene-1,3-diyl)bis-, (E)-
	Terpenoid	Naphthalene, 1,2,3,4-tetrahydro-1-phenyl-
	Phenylpropanoids	4',5-Dihydroxy-7-methoxyflavanone
	Linear Hydrocarbon	Triacontane, 1-bromo-
	Terpenoid	Squalene
	Linear Hydrocarbon	Pentatriacontane
	Others	Eicosanoic acid, 2-(acetyloxy)-1-[(acetyloxy)methyl]ethyl ester
	Others	Butanoic acid, 2-methyl-, ethyl ester
	Phenylpropanoids	Benzene, 1,3-dimethyl-
	Terpenoid	D-Limonene
	Terpenoid	.alpha.-Methyl-.alpha.-[4-methyl-3-pentenyl]oxiranemethanol
	Linear Hydrocarbons	Nonane, 2,5-dimethyl-
	Terpenoid	Linalool
	Terpenoid	Beta-cyclocitral
	Phenylpropanoids	2-Propenoic acid, 3-phenyl-
	Others	Acetophenone, 4'-hydroxy-
	Terpenoid	(1R,3aS,4aS,8aS)-1,4,4,6-Tetramethyl-1,2,3,3a,4,4a,7,8-octahydrocyclopenta[1,4]cyclobuta[1,2]benzene
	Phenylpropanoids	Benzoic acid, 2-methoxy-
	Terpenoid	1H-Benzocyclohepten-7-ol, 2,3,4,4a,5,6,7,8-octahydro-1,1,4a,7-tetramethyl-, cis-
	Terpenoid	.alpha.-Farnesene
	Terpenoid	zerumbone
	Terpenoid	1,6,10-Dodecatrien-3-ol, 3,7,11-trimethyl-, [S-(Z)]-
	Others	10-12-Pentacosadiynoic acid
	Terpenoid	2-(4a,8-Dimethyl-2,3,4,5,6,7-hexahydro-1H-naphthalen-2-yl)propan-2-ol
	Terpenoid	Agarospirol
	Terpenoid	2-Naphthalenemethanol, decahydro-.alpha.,.alpha.,4a-trimethyl-8-methylene-, [2R-(2.alpha.,4a.alpha.,8a.beta.)]-

	Terpenoid	2-Naphthalenemethanol, 1,2,3,4,4a,5,6,8a-octahydro-.alpha.,.alpha.,4a,8-tetramethyl-, [2R-(2.alpha.,4a.alpha.,8a.beta.)]-
	Phenylpropanoids	Benzene, 1,1'-(1,2-cyclobutanediyl)bis-, trans-
	Phenylpropanoids	2-Hexanone, 6-phenyl-
	Phenylpropanoids	Benzene, 1,1'-[1-(2,2-dimethyl-3-butenyl)-1,3-propanediyl]bis-
	Others	3-Methyl-2-butenoic acid, tridec-2-ynyl ester
	Terpenoid	(-)-Spathulenol
	Phenylpropanoids	Benzyl Benzoate
	Linear Hydrocarbons	1-Octadecene
	Phenylpropanoids	Benzoic acid, hept-2-yl ester
BESC-1179	Terpenoid	(1R,4aR,7R,8aR)-7-(2-Hydroxypropan-2-yl)-1,4a-dimethyldecahydronaphthalen-1-ol
	Phenylpropanoids	Benzoic acid, 2-hydroxy-, phenylmethyl ester
	Others	Hexadecanoic acid, methyl ester
	Others	n-Hexadecanoic acid
	Phenylpropanoids	2-Methoxybenzoic acid, benzyl ester
	Phenylpropanoids	2-Methoxybenzyl benzoate
	Phenylpropanoids	3-Phenylpropionyl fluoride-2,2-D2
	Phenylpropanoids	4-Methoxybenzyl benzoate
	Phenylpropanoids	(Z)-Cinnamyl benzoate
	Others	9,12-Octadecadienoic acid (Z,Z)-, methyl ester
	Others	11(Z),14(Z),17(Z)-Eicosatrienoic Acid methyl ester
	Phenylpropanoids	4-(Benzyloxy)-3-methoxybenzoic acid
	Linear Hydrocarbons	Tetracosane
	Others	Eicosanoic acid, methyl ester
	Phenylpropanoids	(-)-Pinostrobin
	Phenylpropanoids	Cinnamyl cinnamate
	Linear Hydrocarbons	Nonacosane
	Phenylpropanoids	2',6'-Dihydroxy 4'-methoxydihydrochalcone, diacetate
	Phenylpropanoids	Benzenepropanoic acid, 3-phenyl-2-propenyl ester
	Phenylpropanoids	5-Hydroxy-4',7-dimethoxyflavanone
	Phenylpropanoids	8-Hydroxy-2,2,5-trimethyl-5-(3-methylbut-2-en-1-yl)-7-(3-phenylpropanoyl)-2H-chromen-6(5H)-one
	Others	Phenol, 4,4'-(3-ethenyl-1-propene-1,3-diyl)bis-, (E)-
	Terpenoid	Naphthalene, 1,2,3,4-tetrahydro-1-phenyl-
	Phenylpropanoids	4',5-Dihydroxy-7-methoxyflavanone
	Others	Pyrrolidine, 2-phenyl-
	Others	Eicosanoic acid, 2,3-bis(acetyloxy)propyl ester
	Others	cis-13-Eicosenoic acid
	Others	(Tetrahydro-2H-pyran-2-yl)methyl 4-methylpentanoate
	Others	Butanoic acid, 2-methyl-, ethyl ester
	Phenylpropanoids	Benzene, 1,3-dimethyl-
	Terpenoid	.alpha.-Methyl-.alpha.-[4-methyl-3-pentenyl]oxiranemethanol
	Terpenoid	Linalool
	Terpenoid	(3R,6S)-2,2,6-Trimethyl-6-vinyltetrahydro-2H-pyran-3-ol
	Terpenoid	beta-cyclocitral
	Others	Acetophenone, 4'-hydroxy-
	Others	2,4-Di-tert-butylphenol
	Terpenoid	1,6,10-Dodecatrien-3-ol, 3,7,11-trimethyl-, [S-(Z)]-
	Terpenoid	Isoaromadendrene epoxide
	Terpenoid	(1R,3E,7E,11R)-1,5,5,8-Tetramethyl-12-oxabicyclo[9.1.0]dodeca-3,7-diene
	Phenylpropanoids	Phenol, 2,4-di-t-butyl-6-nitro-
	Phenylpropanoids	Benzene, 1,1'-(1,2-cyclobutanediyl)bis-, trans-
	Terpenoid	(-)-Spathulenol
	Phenylpropanoids	Benzyl Benzoate
HARC-26-1	Phenylpropanoids	Benzoic acid, hept-2-yl ester
	Phenylpropanoids	Benzoic acid, 2-hydroxy-, phenylmethyl ester
	Phenylpropanoids	2-Methoxybenzoic acid, benzyl ester
	Phenylpropanoids	2-Methoxybenzyl benzoate
	Phenylpropanoids	4-Methoxybenzyl benzoate
	Phenylpropanoids	(E)-Cinnamyl benzoate
	Phenylpropanoids	Benzeneacetic acid, methyl ester
	Phenylpropanoids	4-(Benzyloxy)-3-methoxybenzoic acid
	Others	N,N-dimethylhexadecanamide
	Linear Hydrocarbon	Tetracosane
	Phenylpropanoids	4H-1-Benzopyran-4-one, 2,3-dihydro-5-hydroxy-7-methoxy-2-phenyl-, (S)-
	Phenylpropanoids	2',6'-Dihydroxy 4'-methoxydihydrochalcone, diacetate
	Phenylpropanoids	Benzenepropanoic acid, 3-phenyl-2-propenyl ester
	Linear Hydrocarbon	Nonacosane
	Phenylpropanoids	5-Hydroxy-4',7-dimethoxyflavanone
	Terpenoid	Naphthalene, 1,2,3,4-tetrahydro-1-phenyl-
	Others	Tetratetracontane
	Others	Butanoic acid, 2-methyl-, ethyl ester
	Phenylpropanoids	Benzene, 1,3-dimethyl-
	Terpenoid	D-Limonene
	Terpenoid	.alpha.-Methyl-.alpha.-[4-methyl-3-pentenyl]oxiranemethanol
	Terpenoid	Linalool
	Terpenoid	trans-linalool oxide
	Phenylpropanoids	Benzoic acid
	Terpenoid	(1S)-1,3,3-trimethylnorbornan-2-ol
	Others	Beta-cyclocitral
	Others	4-Vinylphenol
	Linear hydrocarbon	Tetradecane
	Phenylpropanoids	trans-Cinnamic acid
	Phenylpropanoids	Ethanone, 1-(3-hydroxyphenyl)-
	Phenylpropanoids	Acetophenone, 4'-hydroxy-
	Phenylpropanoids	Benzoic acid, 2-methoxy-
	Terpenoid	Naphthalene, decahydro-4a-methyl-1-methylene-7-(1-methylethenyl)-, [4aR-(4a.alpha.,7.alpha.,8a.beta.)]-
	Terpenoid	4.beta.H,5.alpha.-Eremophila-1(10),11-diene
	Terpenoid	1H-Benzocyclohepten-7-ol, 2,3,4,4a,5,6,7,8-octahydro-1,1,4a,7-tetramethyl-, cis-
	Terpenoid	.alpha.-Muurolene
	Terpenoid	.alpha.-Farnesene
	Terpenoid	Naphthalene, 1,2,3,4,4a,5,6,8a-octahydro-7-methyl-4-methylene-1-(1-methylethyl)-, (1.alpha.,4a.beta.,8a.alpha.)-

DENB-17-1	Terpenoid	.delta.-Cadinene
	Terpenoid	1,6,10-Dodecatrien-3-ol, 3,7,11-trimethyl-, [S-(Z)]-
	Terpenoid	Farnesene epoxide, E-
	Terpenoid	4,11-Dimethyl-8-(propan-2-yl)-5,12-dioxatricyclo[9.1.0.04,6]dodecan-7-ol, Ac
	Terpenoid	.alpha.-Cadinol
	Terpenoid	3-Cyclohexene-1-methanol, .alpha.,4-dimethyl-.alpha.-(4-methyl-3-penten
	Phenylpropanoids	Benzene, 1,1'-(1,2-cyclobutanediyl)bis-, trans-
	Terpenoid	trans-Z-.alpha.-Bisabolene epoxide
	Terpenoid	trans-L-Carvyl isobutyrate
	Phenylpropanoids	Benzene, 1,1'-[1-(2,2-dimethyl-3-butenyl)-1,3-propanediyl]bis-
	Others	3-Methyl-2-butenoic acid, tridec-2-ynyl ester
	Terpenoid	(-)-Spathulenol
	Phenylpropanoids	Benzyl Benzoate
	Phenylpropanoids	Benzoic acid, 1-phenylethyl ester
	Phenylpropanoids	Benzoic acid, hept-2-yl ester
	Terpenoid	[(1S,7S,8S,8aS)-8-[2-[(2R,4R)-4-Hydroxy-6-oxooxan-2-yl]ethyl]-7-methyl-1,2,3,4,4a,7,8,8a-octahydronaphthalen-1-yl] (2S)-2-methy
	Phenylpropanoids	Benzoic acid, 2-hydroxy-, phenylmethyl ester
	Linear hydrocarbon	1-Tricosene
	Others	n-Hexadecanoic acid
	Phenylpropanoids	2-Methoxybenzoic acid, benzyl ester
	Phenylpropanoids	2-Methoxybenzyl benzoate
	Phenylpropanoids	4-Methoxybenzyl benzoate
	Phenylpropanoids	(Z)-Cinnamyl benzoate
	Phenylpropanoids	4-(Benzyloxy)-3-methoxybenzoic acid
	Others	N,N-dimethylhexadecanamide
	Linear hydrocarbon	Tetracosane
	Phenylpropanoids	4H-1-Benzopyran-4-one, 2,3-dihydro-5-hydroxy-7-methoxy-2-phenyl-, (S)-
	Phenylpropanoids	Cinnamyl cinnamate
	Others	N,N-Dimethylpalmitamide
	Phenylpropanoids	2',6'-Dihydroxy 4'-methoxydihydrochalcone, diacetate
	Phenylpropanoids	Benzenepropanoic acid, 3-phenyl-2-propenyl ester
	Linear hydrocarbon	Nonacosane
	Phenylpropanoids	5-Hydroxy-4',7-dimethoxyflavanone
	Phenylpropanoids	2,2,5-trimethyl-5-(3-methylbut-2-enyl)-8-oxidanyl-7-(3-phenylpropanoyl)chromen-6-one
	Phenylpropanoids	(E)-hinokiresinol
	Terpenoid	Naphthalene, 1,2,3,4-tetrahydro-1-phenyl-
	Others	Ethanone, 1-[2,3-dihydro-6-hydroxy-2-(1-hydroxy-1-methylethyl)-4-methoxy-7-benzofuranyl]-, (+)-
	Others	4',5-Dihydroxy-7-methoxyflavanone
	Others	Isosakuranetin, diacetate
	Linear hydrocarbon	N-heneicosane
	Phenylpropanoids	Benzoic acid, [(E,E)-3,7,11-trimethyl-2,6,10-dodecatrien-1-yl] ester
	Others	14,16-Hentriacontanedione
	Others	Eicosanoic acid, 2-(acetyloxy)-1-[(acetyloxy)methyl]ethyl ester
	Others	Eicosanoic acid, 2,3-bis(acetyloxy)propyl ester
HOMB-21-3	Others	Butanoic acid, 2-methyl-, ethyl ester
	Phenylpropanoids	Benzene, 1,3-dimethyl-
	Linear hydrocarbons	Undecane
	Terpenoid	beta-cyclocitral
	Phenylpropanoids	2-Propen-1-ol, 3-phenyl-
	Others	Acetophenone, 4'-hydroxy-
	Phenylpropanoids	Benzoic acid, 2-methoxy-
	Phenylpropanoids	Benzene, 1,1'-(1,2-cyclobutanediyl)bis-, trans-
	Phenylpropanoids	Benzyl Benzoate
	Phenylpropanoids	Benzoic acid, 1-phenylethyl ester
	Phenylpropanoids	Benzoic acid, hept-2-yl ester
	Phenylpropanoids	Benzoic acid, 2-hydroxy-, phenylmethyl ester
	Phenylpropanoids	2-Methoxybenzoic acid, benzyl ester
	Phenylpropanoids	2-Methoxybenzyl benzoate
	Phenylpropanoids	(Z)-Cinnamyl benzoate
	Others	Linoleic Acid methyl ester
	Phenylpropanoids	4-(Benzyloxy)-3-methoxybenzoic acid
	Linear hydrocarbons	Tetracosane
	Phenylpropanoids	4H-1-Benzopyran-4-one, 2,3-dihydro-5-hydroxy-7-methoxy-2-phenyl-, (S)-
	Phenylpropanoids	Cinnamyl cinnamate
	Phenylpropanoids	Benzyl trans-4-coumarate
	Linear hydrocarbons	Nonacosane
	Phenylpropanoids	2',6'-Dihydroxy 4'-methoxydihydrochalcone, diacetate
	Phenylpropanoids	Benzenepropanoic acid, 3-phenyl-2-propenyl ester
	Phenylpropanoids	5-Hydroxy-4',7-dimethoxyflavanone
	Phenylpropanoids	4',5-Dihydroxy-7-methoxyflavanone
	Others	Pyrrolidine, 2-phenyl-
	Others	Butanoic acid, 2-methyl-, ethyl ester
	Phenylpropanoids	Benzene, 1,3-dimethyl-
	Terpenoid	Linalool
	Linear hydrocarbons	Dodecane
	Terpenoid	1-Cyclohexene-1-carboxaldehyde, 2,6,6-trimethyl-
	Terpenoid	trans-.alpha.-Bergamotene
	Others	Acetophenone, 4'-hydroxy-
	Phenylpropanoids	Benzoic acid, 2-methoxy-
	Terpenoid	Italicene
	Terpenoid	.gamma.-Muurolene
	Terpenoid	.delta.-Cadinene
	Terpenoid	1,6,10-Dodecatrien-3-ol, 3,7,11-trimethyl-, [S-(Z)]-
	Terpenoid	gamma-ionone
	Terpenoid	(-)-α-bisabolol oxide B
	Terpenoid	beta-bisabolol
	Terpenoid	6-Methyl-2-(3-methyl-1-cyclohex-3-enyl)-5-hepten-2-ol
	Phenylpropanoids	Benzene, 1,1'-(1,2-cyclobutanediyl)bis-, trans-
	Phenylpropanoids	[3,3-Dimethyl-1-(2-phenylethyl)-4-pentenyl]benzene
	Others	Tetradecanoic acid

BESC-102	Phenylpropanoids	Benzyl Benzoate
	Terpenoid	4-(2,2,6-Trimethylcyclohexyl)-2-butanone
	Phenylpropanoids	Benzoic acid, 1-phenylethyl ester
	Phenylpropanoids	Benzoic acid, hept-2-yl ester
	Phenylpropanoids	Benzoic acid, 2-hydroxy-, phenylmethyl ester
	Others	n-Hexadecanoic acid
	Phenylpropanoids	2-Methoxybenzoic acid, benzyl ester
	Phenylpropanoids	2-Methoxybenzyl benzoate
	Phenylpropanoids	(Z)-Cinnamyl benzoate
	Linear hydrocarbons	Eicosane
	Linear hydrocarbons	Tetracosane
	Phenylpropanoids	4H-1-Benzopyran-4-one, 2,3-dihydro-5-hydroxy-7-methoxy-2-phenyl-, (S)-
	Phenylpropanoids	2',6'-Dihydroxy 4'-methoxydihydrochalcone, diacetate
	Phenylpropanoids	Benzenepropanoic acid, 3-phenyl-2-propenyl ester
	Phenylpropanoids	5-Hydroxy-4',7-dimethoxyflavanone
	Terpenoid	Naphthalene, 1,2,3,4-tetrahydro-1-phenyl-
	Phenylpropanoids	4',5-Dihydroxy-7-methoxyflavanone
	Linear hydrocarbons	Hexatriacontane
	Terpenoid	Squalene
	Linear hydrocarbons	Pentatriacontane
	Others	Cholesterol
GW-7091	Others	Acetic acid, butyl ester
	Others	Butanoic acid, 2-methyl-, ethyl ester
	Others	Acetophenone, 4'-hydroxy-
	Terpenoid	1,6,10-Dodecatrien-3-ol, 3,7,11-trimethyl-, [S-(Z)]-
	Terpenoid	2-Naphthalenemethanol, 1,2,3,4,4a,5,6,7-octahydro-.alpha.,.alpha.,4a,8-tetramethyl-, (2R-cis)-
	Terpenoid	2-Naphthalenemethanol, decahydro-.alpha.,.alpha.,4a-trimethyl-8-methylene-, [2R-(2.alpha.,4a.alpha.,8a.beta.)]-
	Terpenoid	2-Naphthalenemethanol, 1,2,3,4,4a,5,6,8a-octahydro-.alpha.,.alpha.,4a,8-tetramethyl-, [2R-(2.alpha.,4a.alpha.,8a.beta.)]-
	Phenylpropanoids	Benzene, 1,1'-(1,2-cyclobutanediyl)bis-, trans-
	Phenylpropanoids	Benzyl Benzoate
	Terpenoid	cryptomeridiol
	Phenylpropanoids	Benzoic acid, 2-hydroxy-, phenylmethyl ester
	Phenylpropanoids	2-Methoxybenzoic acid, benzyl ester
	Phenylpropanoids	(Z)-Cinnamyl benzoate
	Linear hydrocarbons	Tetracosane
	Phenylpropanoids	pinostrobin chalcone
	Phenylpropanoids	Cinnamyl cinnamate
	Phenylpropanoids	2',6'-Dihydroxy 4'-methoxydihydrochalcone, diacetate
	Phenylpropanoids	Benzenepropanoic acid, 3-phenyl-2-propenyl ester
	Linear hydrocarbons	Nonacosane
	Phenylpropanoids	5-Hydroxy-4',7-dimethoxyflavanone
	Terpenoid	Naphthalene, 1,2,3,4-tetrahydro-1-phenyl-
GW-7094	Others	Acetic acid ethyl ester
	Others	4-Vinylphenol
	Others	Acetophenone, 4'-hydroxy-
	Terpenoid	1,6,10-Dodecatrien-3-ol, 3,7,11-trimethyl-, (E)-
	Phenylpropanoids	Benzyl Benzoate
	Phenylpropanoids	Benzoic acid, 2-hydroxy-, phenylmethyl ester
	Phenylpropanoids	2-Methoxybenzoic acid, benzyl ester
	Linear hydrocarbons	Dodecane, 2-methyl-
	Phenylpropanoids	2-Propen-1-one, 1-(2,6-dihydroxy-4-methoxyphenyl)-3-phenyl-, (E)-
	Linear hydrocarbons	N-heneicosane
	Phenylpropanoids	2',6'-Dihydroxy 4'-methoxydihydrochalcone, diacetate
BESC-291	Others	Butanoic acid, 2-methyl-, ethyl ester
	Phenylpropanoids	Benzene, 1,3-dimethyl-
	Terpenoid	D-Limonene
	Linear hydrocarbons	Undecane
	Others	1-Cyclohexene-1-carboxaldehyde, 2,6,6-trimethyl-
	Phenylpropanoids	trans-Cinnamic acid
	Others	Acetophenone, 4'-hydroxy-
	Terpenoid	Naphthalene, 1,2,3,4,4a,5,6,8a-octahydro-7-methyl-4-methylene-1-(1-methylethyl)-, (1.alpha.,4a.beta.,8a.alpha.)-
	Phenylpropanoids	Benzoic acid, 2-methoxy-
	Terpenoid	1H-Benzocyclohepten-7-ol, 2,3,4,4a,5,6,7,8-octahydro-1,1,4a,7-tetramethyl-, cis-
	Terpenoid	Bicyclogermacrene
	Terpenoid	.delta.-Cadinene
	Terpenoid	1,6,10-Dodecatrien-3-ol, 3,7,11-trimethyl-, [S-(Z)]-
	Terpenoid	Farnesene epoxide, E-
	Terpenoid	2-Naphthalenemethanol, 1,2,3,4,4a,5,6,7-octahydro-.alpha.,.alpha.,4a,8-tetramethyl-, (2R-cis)-
	Terpenoid	Guaiol
	Terpenoid	2-Naphthalenemethanol, decahydro-.alpha.,.alpha.,4a-trimethyl-8-methylene-, [2R-(2.alpha.,4a.alpha.,8a.beta.)]-
	Terpenoid	(-)-10-epi.gamma.-Eudsemol
	Terpenoid	3-Cyclohexene-1-methanol, .alpha.,4-dimethyl-.alpha.-(4-methyl-3-penten
	Terpenoid	2,6,10-Dodecatrien-1-ol, 3,7,11-trimethyl-
	Others	3-Methyl-2-butenoic acid, tridec-2-ynyl ester
	Terpenoid	(-)-Spathulenol
	Phenylpropanoids	Benzyl Benzoate
	Terpenoid	(1R,4aR,7R,8aR)-7-(2-Hydroxypropan-2-yl)-1,4a-dimethyldecahydronaphthalen-1-ol
	Phenylpropanoids	Benzoic acid, hept-2-yl ester
	Terpenoid	Boscartol F
	Terpenoid	Neophytadiene
	Phenylpropanoids	Benzoic acid, 2-hydroxy-, phenylmethyl ester
	Phenylpropanoids	2-Methoxybenzoic acid, benzyl ester
	Phenylpropanoids	2-Methoxybenzyl benzoate
	Phenylpropanoids	(Z)-Cinnamyl benzoate
	Phenylpropanoids	Benzeneacetic acid, methyl ester
	Phenylpropanoids	4-(Benzyloxy)-3-methoxybenzoic acid
	Phenylpropanoids	4-(Benzyloxy)-2-nitroaniline
	Linear hydrocarbons	N-heneicosane
	Linear hydrocarbons	Tetracosane
	Phenylpropanoids	2-Propen-1-one, 1-(2,6-dihydroxy-4-methoxyphenyl)-3-phenyl-, (E)-

	Phenylpropanoids	Cinnamyl cinnamate
	Phenylpropanoids	2',6'-Dihydroxy 4'-methoxydihydrochalcone, diacetate
	Phenylpropanoids	Benzenepropanoic acid, 3-phenyl-2-propenyl ester
	Linear hydrocarbons	Nonacosane
	Phenylpropanoids	5-Hydroxy-4',7-dimethoxyflavanone
	Phenylpropanoids	8-Hydroxy-2,2,5-trimethyl-5-(3-methylbut-2-en-1-yl)-7-(3-phenylpropanoyl)-2H-chromen-6(5H)-one
GW-4583	Others	2,4-Dimethyl-1-heptene
	Others	Butanoic acid, 2-methyl-, ethyl ester
	Phenylpropanoids	Benzene, 1,3-dimethyl-
	Linear hydrocarbons	Decane
	Linear hydrocarbons	Undecane
	Linear hydrocarbons	Dodecane
	Linear hydrocarbons	Tetradecane
	Phenylpropanoids	trans-Cinnamic acid
	Others	Acetophenone, 4'-hydroxy-
	Others	2,4-Di-tert-butylphenol
	Terpenoid	1,6,10-Dodecatrien-3-ol, 3,7,11-trimethyl-, (E)-
	Terpenoid	trans-Z-.alpha.-Bisabolene epoxide
	Terpenoid	2-(4a,8-Dimethyl-2,3,4,5,6,7-hexahydro-1H-naphthalen-2-yl)propan-2-ol
	Terpenoid	Agarospirol
	Terpenoid	2-Naphthalenemethanol, decahydro-.alpha.,.alpha.,4a-trimethyl-8-methylene-, [2R-(2.alpha.,4a.alpha.,8a.beta.)]-
	Terpenoid	2-Naphthalenemethanol, 1,2,3,4,4a,5,6,8a-octahydro-.alpha.,.alpha.,4a,8-tetramethyl-, [2R-(2.alpha.,4a.alpha.,8a.beta.)]-
	Others	Phenol, 2,4-di-t-butyl-6-nitro-
	Phenylpropanoids	Benzyl Benzoate
	Terpenoid	cryptomeridiol
	Phenylpropanoids	Benzoic acid, 2-hydroxy-, phenylmethyl ester
	Phenylpropanoids	2-Methoxybenzoic acid, benzyl ester
	Linear hydrocarbons	Eicosane
	Linear hydrocarbons	Nonacosane
	Linear hydrocarbons	Tetracosane
	Others	5-Hydroxy-4',7-dimethoxyflavanone
DENA-17-3	Others	Butanoic acid, 2-methyl-, ethyl ester
	Phenylpropanoids	Benzene, 1,3-dimethyl-
	Linear hydrocarbons	Decane
	Others	2-Cyclohexyl-hex-5-en-2-ol
	Linear hydrocarbons	Undecane
	Linear hydrocarbons	Dodecane
	Linear hydrocarbons	Tetradecane
	Others	Acetophenone, 4'-hydroxy-
	Phenylpropanoids	1-Ethyl-4-methoxybenzene
	Phenylpropanoids	(p-Hydroxyphenyl)glyoxal
	Others	2,4-Di-tert-butylphenol
	Terpenoid	.delta.-Cadinene
	Terpenoid	1,6,10-Dodecatrien-3-ol, 3,7,11-trimethyl-, [S-(Z)]-
	Linear hydrocarbons	Octadecane, 1-chloro-
	Terpenoid	2-Naphthalenemethanol, decahydro-.alpha.,.alpha.,4a-trimethyl-8-methylene-, [2R-(2.alpha.,4a.alpha.,8a.beta.)]-
	Terpenoid	(-)-10-epi.gamma.-Eudsemol
	Others	Phenol, 2,4-di-t-butyl-6-nitro-
	Phenylpropanoids	Benzyl Benzoate
	Phenylpropanoids	Benzoic acid, 1-phenylethyl ester
	Phenylpropanoids	3-Hexen-1-ol benzoate
	Phenylpropanoids	Benzoic acid, 2-hydroxy-, phenylmethyl ester
	Phenylpropanoids	2-Methoxybenzoic acid, benzyl ester
	Linear hydrocarbons	Eicosane
	Linear hydrocarbons	N-heneicosane
	Phenylpropanoids	pinostrobin chalcone
	Linear hydrocarbons	Tetracosane
	Phenylpropanoids	2',6'-Dihydroxy 4'-methoxydihydrochalcone, diacetate
	Phenylpropanoids	Benzenepropanoic acid, 3-phenyl-2-propenyl ester
	Linear hydrocarbons	Nonacosane
	Phenylpropanoids	5-Hydroxy-4',7-dimethoxyflavanone
GW-4584	Others	Butanoic acid, 2-methyl-, ethyl ester
	Phenylpropanoids	Benzene, 1,3-dimethyl-
	Linear Hydrocarbons	Undecane
	Linear Hydrocarbons	Dodecane
	Linear Hydrocarbons	Hexadecane
	Phenylpropanoids	trans-Cinnamic acid
	Others	Acetophenone, 4'-hydroxy-
	Others	2,4-Di-tert-butylphenol
	Terpenoid	1,6,10-Dodecatrien-3-ol, 3,7,11-trimethyl-, [S-(Z)]-
	Terpenoid	trans-Z-.alpha.-Bisabolene epoxide
	Phenylpropanoids	Benzyl Benzoate
	Phenylpropanoids	Benzoic acid, 2-hydroxy-, phenylmethyl ester
	Phenylpropanoids	2-Methoxybenzoic acid, benzyl ester
	Phenylpropanoids	(Z)-Cinnamyl benzoate
	Linear Hydrocarbons	Eicosane
	Phenylpropanoids	pinostrobin chalcone
	Phenylpropanoids	Cinnamyl cinnamate
	Linear Hydrocarbons	N-heneicosane
	Phenylpropanoids	2',6'-Dihydroxy 4'-methoxydihydrochalcone, diacetate
	Phenylpropanoids	Benzenepropanoic acid, 3-phenyl-2-propenyl ester
	Linear Hydrocarbons	Tetracosane
	Phenylpropanoids	5-Hydroxy-4',7-dimethoxyflavanone
	Others	Butanoic acid, 2-methyl-, ethyl ester
	Phenylpropanoids	Benzene, 1,3-dimethyl-
	Linear hydrocarbons	Undecane
	Phenylpropanoids	trans-Cinnamic acid
	Others	Acetophenone, 4'-hydroxy-
	Terpenoid	1H-Benzocyclohepten-7-ol, 2,3,4,4a,5,6,7,8-octahydro-1,1,4a,7-tetramethyl-, cis-
	Terpenoid	1,6,10-Dodecatrien-3-ol, 3,7,11-trimethyl-, (E)-

BESC-855	Terpenoid	Farnesene epoxide, E-
	Phenylpropanoids	Benzyl Benzoate
	Terpenoid	2-Cyclohexen-1-one, 2-methyl-5-(1-methylethenyl)-
	Phenylpropanoids	Benzoic acid, 2-hydroxy-, phenylmethyl ester
	Phenylpropanoids	2-Methoxybenzoic acid, benzyl ester
	Phenylpropanoids	(Z)-Cinnamyl benzoate
	Linear hydrocarbons	N-heneicosane
	Linear hydrocarbons	Eicosane
	Phenylpropanoids	2-Propen-1-one, 1-(2,6-dihydroxy-4-methoxyphenyl)-3-phenyl-, (E)-
	Phenylpropanoids	Cinnamyl cinnamate
	Linear hydrocarbons	Tetracosane
	Phenylpropanoids	2',6'-Dihydroxy 4'-methoxydihydrochalcone, diacetate
	Phenylpropanoids	Benzenepropanoic acid, 3-phenyl-2-propenyl ester
	Linear hydrocarbons	Nonacosane
	Phenylpropanoids	5-Hydroxy-4',7-dimethoxyflavanone
BESC-1070	Others	Butanoic acid, 2-methyl-, ethyl ester
	Phenylpropanoids	Benzene, 1,3-dimethyl-
	Terpenoid	2-Pinene
	Linear hydrocarbons	Decane
	Terpenoid	D-Limonene
	Others	2-Pyrrolidinone, 1-(2-aminoethyl)-
	Terpenoid	Linalool
	Others	Nonanal
	Terpenoid	.alpha.-Terpineol
	Linear hydrocarbons	Dodecane
	Linear hydrocarbons	Tetradecane
	Others	Acetophenone, 4'-hydroxy-
	Others	2,4-Di-tert-butylphenol
	Terpenoid	1,6,10-Dodecatrien-3-ol, 3,7,11-trimethyl-, (E)-
	Terpenoid	Bergamotol, Z-.alpha.-trans-
	Others	Phenol, 2,4-di-t-butyl-6-nitro-
	Phenylpropanoids	Benzyl Benzoate
	Phenylpropanoids	Benzoic acid, 1-phenylethyl ester
	Phenylpropanoids	3-Hexen-1-ol benzoate
	Phenylpropanoids	Benzoic acid, 2-hydroxy-, phenylmethyl ester
	Phenylpropanoids	2-Methoxybenzoic acid, benzyl ester
	Linear hydrocarbons	N-heneicosane
	Linear hydrocarbons	Eicosane
	Phenylpropanoids	pinostrobin chalcone
	Linear hydrocarbons	Nonacosane
	Phenylpropanoids	2',6'-Dihydroxy 4'-methoxydihydrochalcone, diacetate
	Phenylpropanoids	Benzenepropanoic acid, 3-phenyl-2-propenyl ester
	Phenylpropanoids	5-Hydroxy-4',7-dimethoxyflavanone
BESC-290	Others	Butanoic acid, 2-methyl-, ethyl ester
	Others	2-Butanone
	Phenylpropanoids	Benzene, 1,3-dimethyl-
	Others	1,3,5,7-Cyclooctatetraene
	Linear hydrocarbons	Decane
	Linear hydrocarbons	Undecane
	Linear hydrocarbons	Dodecane
	Linear hydrocarbons	Tetradecane
	Phenylpropanoids	trans-Cinnamic acid
	Others	Acetophenone, 4'-hydroxy-
	Others	2,4-Di-tert-butylphenol
	Terpenoid	1,6,10-Dodecatrien-3-ol, 3,7,11-trimethyl-, [S-(Z)]-
	Linear hydrocarbons	1-Octadecanesulphonyl chloride
	Terpenoid	2-Naphthalenemethanol, decahydro-.alpha.,.alpha.,4a-trimethyl-8-methylene-, [2R-(2.alpha.,4a.alpha.,8a.beta.)]-
	Terpenoid	2-Naphthalenemethanol, 1,2,3,4,4a,5,6,8a-octahydro-.alpha.,.alpha.,4a,8-tetramethyl-, [2R-(2.alpha.,4a.alpha.,8a.beta.)]-
	Others	Phenol, 2,4-di-t-butyl-6-nitro-
	Phenylpropanoids	Benzyl Benzoate
	Phenylpropanoids	Benzoic acid, 1-phenylethyl ester
	Phenylpropanoids	Benzoic acid, hept-2-yl ester
	Terpenoid	cryptomeridiol
	Phenylpropanoids	Benzoic acid, 2-hydroxy-, phenylmethyl ester
	Others	n-Hexadecanoic acid
	Phenylpropanoids	2-Methoxybenzoic acid, benzyl ester
	Phenylpropanoids	2-Methoxybenzyl benzoate
	Phenylpropanoids	(E)-Cinnamyl benzoate
	Linear hydrocarbons	Tetracosane
	Phenylpropanoids	2-Phenylpropionsaeure
	Others	Acetic acid n-octadecyl ester
	Phenylpropanoids	Pinostrobin chalcone
	Phenylpropanoids	Cinnamyl cinnamate
	Linear hydrocarbons	Nonacosane
	Phenylpropanoids	2',6'-Dihydroxy 4'-methoxydihydrochalcone, diacetate
	Phenylpropanoids	Benzenepropanoic acid, 3-phenyl-2-propenyl ester
	Phenylpropanoids	5-Hydroxy-4',7-dimethoxyflavanone
CHWJ-25-6	Others	Butanoic acid, 2-methyl-, ethyl ester
	Others	Acetophenone, 4'-hydroxy-
	Others	2,4-Di-tert-butylphenol
	Terpenoid	1,6,10-Dodecatrien-3-ol, 3,7,11-trimethyl-, [S-(Z)]-
	Phenylpropanoids	Benzyl Benzoate
	Phenylpropanoids	Benzoic acid, 2-hydroxy-, phenylmethyl ester
	Phenylpropanoids	2-Methoxybenzoic acid, benzyl ester
	Phenylpropanoids	(Z)-Cinnamyl benzoate
	Linear hydrocarbons	Tetracosane
	Phenylpropanoids	pinostrobin chalcone
	Phenylpropanoids	Cinnamyl cinnamate
	Linear hydrocarbons	Nonacosane
	Phenylpropanoids	2',6'-Dihydroxy 4'-methoxydihydrochalcone, diacetate

	Phenylpropanoids	Benzenepropanoic acid, 3-phenyl-2-propenyl ester
	Phenylpropanoids	5-Hydroxy-4',7-dimethoxyflavanone
SKWC-24-1	Others	Butanoic acid, 2-methyl-, ethyl ester
	Others	Butanoic acid, 3-methyl-, ethyl ester
	Phenylpropanoids	Benzene, 1,3-dimethyl-
	Others	Heptane, 2,2,4,6,6-pentamethyl-
	Linear hydrocarbons	Decane
	Linear hydrocarbons	Undecane
	Linear hydrocarbons	Dodecane
	Linear hydrocarbons	Tetradecane
	Phenylpropanoids	trans-Cinnamic acid
	Others	Acetophenone, 4'-hydroxy-
	Terpenoid	1H-Benzocyclohepten-7-ol, 2,3,4,4a,5,6,7,8-octahydro-1,1,4a,7-tetramethyl-, cis-
	Terpenoid	1,6,10-Dodecatrien-3-ol, 3,7,11-trimethyl-, [S-(Z)]-
	Phenylpropanoids	Benzene, 1,1'-(1,2-cyclobutanediyl)bis-, trans-
	Phenylpropanoids	Benzyl Benzoate
	Phenylpropanoids	Benzoic acid, hept-2-yl ester
	Phenylpropanoids	Benzoic acid, 2-hydroxy-, phenylmethyl ester
	Phenylpropanoids	2-Methoxybenzoic acid, benzyl ester
	Phenylpropanoids	2-Methoxybenzyl benzoate
	Phenylpropanoids	(Z)-Cinnamyl benzoate
	Phenylpropanoids	Benzenemethanol, 2-hydroxy-3,6-dimethyl-4-(phenylmethoxy)-
	Phenylpropanoids	4-(Benzyloxy)-2-nitroaniline
	Linear hydrocarbons	Tetracosane
	Phenylpropanoids	4H-1-Benzopyran-4-one, 2,3-dihydro-5-hydroxy-7-methoxy-2-phenyl-, (S)-
	Linear hydrocarbons	Eicosane
	Linear hydrocarbons	Hexatriacontane
	Phenylpropanoids	2',6'-Dihydroxy 4'-methoxydihydrochalcone, diacetate
	Phenylpropanoids	Benzenepropanoic acid, 3-phenyl-2-propenyl ester
	Phenylpropanoids	Benzofuran-6-ol-3-one, 2-(4-ethoxycarbonyl)benzylidene-
	Others	2',4',6'-Trihydroxydihydrochalcone
	Linear hydrocarbons	Tricosane
	Others	5-Hydroxy-4',7-dimethoxyflavanone
	Others	Phenol, 4,4'-(3-ethenyl-1-propene-1,3-diyl)bis-, (E)-
	Terpenoid	Naphthalene, 1,2,3,4-tetrahydro-1-phenyl-
	Others	2-Hexyldodecyl acetate
	Linear hydrocarbons	2-Methylhexacosane
LILD-26-5	Others	Butanoic acid, 2-methyl-, ethyl ester
	Others	Butanoic acid, 3-methyl-, ethyl ester
	Phenylpropanoids	Benzene, 1,3-dimethyl-
	Others	Heptane, 2,2,4,6,6-pentamethyl-
	Linear hydrocarbons	Undecane
	Linear hydrocarbons	Dodecane
	Linear hydrocarbons	Tetradecane
	Others	Acetophenone, 4'-hydroxy-
	Terpenoid	1H-Benzocyclohepten-7-ol, 2,3,4,4a,5,6,7,8-octahydro-1,1,4a,7-tetramethyl-, cis-
	Terpenoid	1,6,10-Dodecatrien-3-ol, 3,7,11-trimethyl-, [S-(Z)]-
	Phenylpropanoids	Benzene, 1,1'-(1,2-cyclobutanediyl)bis-, trans-
	Phenylpropanoids	Benzyl Benzoate
	Phenylpropanoids	Benzoic acid, hept-2-yl ester
	Phenylpropanoids	Benzoic acid, 2-hydroxy-, phenylmethyl ester
	Phenylpropanoids	2-Methoxybenzoic acid, benzyl ester
	Phenylpropanoids	2-Methoxybenzyl benzoate
	Phenylpropanoids	(Z)-Cinnamyl benzoate
	Phenylpropanoids	Benzenemethanol, 2-hydroxy-3,6-dimethyl-4-(phenylmethoxy)-
	Linear hydrocarbons	N-heneicosane
	Linear hydrocarbons	Tetracosane
	Phenylpropanoids	4H-1-Benzopyran-4-one, 2,3-dihydro-5-hydroxy-7-methoxy-2-phenyl-, (S)-
	Phenylpropanoids	2',6'-Dihydroxy 4'-methoxydihydrochalcone, diacetate
	Phenylpropanoids	Benzenepropanoic acid, 3-phenyl-2-propenyl ester
	Phenylpropanoids	2',4',6'-Trihydroxydihydrochalcone
	Linear hydrocarbons	Tricosane
	Others	5-Hydroxy-4',7-dimethoxyflavanone
	Others	Phenol, 4,4'-(3-ethenyl-1-propene-1,3-diyl)bis-, (E)-
	Terpenoid	Naphthalene, 1,2,3,4-tetrahydro-1-phenyl-
	Linear hydrocarbons	Pentatriacontane
WELC-24-5	Others	Butanoic acid, 2-methyl-, ethyl ester
	Others	Butanoic acid, 3-methyl-, ethyl ester
	Phenylpropanoids	Benzene, 1,3-dimethyl-
	Others	Heptane, 2,2,4,6,6-pentamethyl-
	Linear hydrocarbons	Undecane
	Linear hydrocarbons	Dodecane
	Linear hydrocarbons	Tetradecane
	Others	Acetophenone, 4'-hydroxy-
	Terpenoid	1H-Benzocyclohepten-7-ol, 2,3,4,4a,5,6,7,8-octahydro-1,1,4a,7-tetramethyl-, cis-
	Terpenoid	1,6,10-Dodecatrien-3-ol, 3,7,11-trimethyl-, [S-(Z)]-
	Phenylpropanoids	Benzene, 1,1'-(1,2-cyclobutanediyl)bis-, trans-
	Phenylpropanoids	Benzyl Benzoate
	Phenylpropanoids	Benzoic acid, hept-2-yl ester
	Phenylpropanoids	Benzoic acid, 2-hydroxy-, phenylmethyl ester
	Phenylpropanoids	2-Methoxybenzoic acid, benzyl ester
	Phenylpropanoids	2-Methoxybenzyl benzoate
	Phenylpropanoids	(Z)-Cinnamyl benzoate
	Phenylpropanoids	2-Phenylpropionsaeure
	Linear hydrocarbons	Tetracosane
	Phenylpropanoids	4H-1-Benzopyran-4-one, 2,3-dihydro-5-hydroxy-7-methoxy-2-phenyl-, (S)-
	Others	2',6'-Dihydroxy 4'-methoxydihydrochalcone, diacetate
	Phenylpropanoids	Benzenepropanoic acid, 3-phenyl-2-propenyl ester
	Linear hydrocarbons	Tricosane
	Others	Phenol, 4,4'-(3-ethenyl-1-propene-1,3-diyl)bis-, (E)-

	Others	1-Heptacosanol
	Terpenoid	Naphthalene, 1,2,3,4-tetrahydro-1-phenyl-
	Linear hydrocarbons	Pentatriacontane
BESC-136	Others	Butanoic acid, 2-methyl-, ethyl ester
	Others	Butanoic acid, 3-methyl-, ethyl ester
	Phenylpropanoids	Benzene, 1,3-dimethyl-
	Others	Heptane, 2,2,4,6,6-pentamethyl-
	Linear hydrocarbons	Undecane
	Linear hydrocarbons	Dodecane
	Linear hydrocarbons	Tetradecane
	Others	Acetophenone, 4'-hydroxy-
	Terpenoid	1H-Benzocyclohepten-7-ol, 2,3,4,4a,5,6,7,8-octahydro-1,1,4a,7-tetramethyl-, cis-
	Terpenoid	1,6,10-Dodecatrien-3-ol, 3,7,11-trimethyl-, [S-(Z)]-
	Phenylpropanoids	Benzene, 1,1'-(1,2-cyclobutanediyl)bis-, trans-
	Phenylpropanoids	Benzyl Benzoate
	Phenylpropanoids	Benzoic acid, hept-2-yl ester
	Phenylpropanoids	Benzoic acid, 2-hydroxy-, phenylmethyl ester
	Phenylpropanoids	2-Methoxybenzoic acid, benzyl ester
	Phenylpropanoids	2-Methoxybenzyl benzoate
	Phenylpropanoids	(Z)-Cinnamyl benzoate
	Phenylpropanoids	Benzenemethanol, 2-hydroxy-3,6-dimethyl-4-(phenylmethoxy)-
	Linear hydrocarbons	Tetracosane
	Linear hydrocarbons	Eicosane
	Phenylpropanoids	(-)-Pinostrobin
	Linear hydrocarbons	Hexatriacontane
	Phenylpropanoids	2',6'-Dihydroxy 4'-methoxydihydrochalcone, diacetate
	Phenylpropanoids	Benzenepropanoic acid, 3-phenyl-2-propenyl ester
	Linear hydrocarbons	Tricosane
	Others	norlignan
	Phenylpropanoids	Naphthalene, 1,2,3,4-tetrahydro-1-phenyl-
	Linear hydrocarbons	Pentatriacontane
SLMB-28-3	Others	Butanoic acid, 2-methyl-, ethyl ester
	Phenylpropanoids	Benzene, 1,3-dimethyl-
	Others	Heptane, 2,2,4,6,6-pentamethyl-
	Linear hydrocarbons	Undecane
	Linear hydrocarbons	Dodecane
	Linear hydrocarbons	Tetradecane
	Others	Acetophenone, 4'-hydroxy-
	Terpenoid	1H-Benzocyclohepten-7-ol, 2,3,4,4a,5,6,7,8-octahydro-1,1,4a,7-tetramethyl-, cis-
	Terpenoid	1,6,10-Dodecatrien-3-ol, 3,7,11-trimethyl-, [S-(Z)]-
	Phenylpropanoids	Benzene, 1,1'-(1,2-cyclobutanediyl)bis-, trans-
	Phenylpropanoids	Benzyl Benzoate
	Phenylpropanoids	Benzoic acid, hept-2-yl ester
	Phenylpropanoids	Benzoic acid, 2-hydroxy-, phenylmethyl ester
	Phenylpropanoids	2-Methoxybenzoic acid, benzyl ester
	Phenylpropanoids	2-Methoxybenzyl benzoate
	Phenylpropanoids	(Z)-Cinnamyl benzoate
	Linear hydrocarbons	Hexacosane
	Linear hydrocarbons	Tetracosane
	Phenylpropanoids	4H-1-Benzopyran-4-one, 2,3-dihydro-5-hydroxy-7-methoxy-2-phenyl-, (S)-
	Phenylpropanoids	2',6'-Dihydroxy 4'-methoxydihydrochalcone, diacetate
	Phenylpropanoids	Benzenepropanoic acid, 3-phenyl-2-propenyl ester
	Linear hydrocarbons	Tricosane
	Others	Phenol, 4,4'-(3-ethenyl-1-propene-1,3-diyl)bis-, (E)-
	Terpenoid	Naphthalene, 1,2,3,4-tetrahydro-1-phenyl-
	Others	Pentatriacontane
SQMB-25-3	Others	Butanoic acid, 2-methyl-, ethyl ester
	Phenylpropanoids	Benzene, 1,3-dimethyl-
	Others	Heptane, 2,2,4,6,6-pentamethyl-
	Terpenoid	D-Limonene
	Linear hydrocarbons	Undecane
	Phenylpropanoids	2-Propenoic acid, 3-phenyl-
	Others	Acetophenone, 4'-hydroxy-
	Terpenoid	1H-Benzocyclohepten-7-ol, 2,3,4,4a,5,6,7,8-octahydro-1,1,4a,7-tetramethyl-, cis-
	Terpenoid	1,6,10-Dodecatrien-3-ol, 3,7,11-trimethyl-, [S-(Z)]-
	Terpenoid	1.6-Farnesadiene-3.10.11-triol, acetate
	Phenylpropanoids	Benzene, 1,1'-(1,2-cyclobutanediyl)bis-, trans-
	Phenylpropanoids	Benzyl Benzoate
	Phenylpropanoids	2-Methoxybenzoic acid, benzyl ester
	Phenylpropanoids	2-Methoxybenzyl benzoate
	Linear hydrocarbons	Tetracosane
	Linear hydrocarbons	Tetratriacontane
	Linear hydrocarbons	Tricosane
	Others	4',5-Dihydroxy-7-methoxyflavanone
	Others	Butanoic acid, 2-methyl-, ethyl ester
	Phenylpropanoids	Benzene, 1,3-dimethyl-
	Terpenoid	Linalool
	Others	4-Vinylphenol
	Phenylpropanoids	2-Propen-1-ol, 3-phenyl-
	Phenylpropanoids	Benzoic acid, 2-methoxy-, methyl ester
	Others	Acetophenone, 4'-hydroxy-
	Phenylpropanoids	Benzoic acid, 2-methoxy-
	Terpenoid	1H-Benzocyclohepten-7-ol, 2,3,4,4a,5,6,7,8-octahydro-1,1,4a,7-tetramethyl-, cis-
	Terpenoid	1,6,10-Dodecatrien-3-ol, 3,7,11-trimethyl-, [S-(Z)]-
	Terpenoid	(1R,3E,7E,11R)-1,5,5,8-Tetramethyl-12-oxabicyclo[9.1.0]dodeca-3,7-diene
	Phenylpropanoids	Benzyl Benzoate
	Phenylpropanoids	Benzoic acid, 1-phenylethyl ester
	Phenylpropanoids	Benzoic acid, hept-2-yl ester
	Phenylpropanoids	Benzoic acid, 2-hydroxy-, phenylmethyl ester
	Linear hydrocarbons	Isoheptadecanol

GW-9882	Phenylpropanoids	2-Methoxybenzoic acid, benzyl ester
	Phenylpropanoids	2-Methoxybenzyl benzoate
	Phenylpropanoids	(Z)-Cinnamyl benzoate
	Phenylpropanoids	Benzenemethanol, 2-hydroxy-3,6-dimethyl-4-(phenylmethoxy)-
	Phenylpropanoids	4-(Benzyloxy)-2-nitroaniline
	Linear hydrocarbons	Tetracosane
	Phenylpropanoids	2-Propen-1-one, 1-(2,6-dihydroxy-4-methoxyphenyl)-3-phenyl-, (E)-
	Phenylpropanoids	Cinnamyl cinnamate
	Others	2',6'-Dihydroxy 4'-methoxydihydrochalcone, diacetate
	Phenylpropanoids	Benzenepropanoic acid, 3-phenyl-2-propenyl ester
	Linear hydrocarbons	Pentatriacontane
	Others	5-Hydroxy-4',7-dimethoxyflavanone
	Others	norlignan
	Others	1-Hexadecanol, 2-methyl-
	Others	4',5-Dihydroxy-7-methoxyflavanone
	Phenylpropanoids	Isosakuranetin, diacetate
	Others	Eicosyl nonyl ether
	Others	2H-Indeno[1,2-b]furan-2-one, 3,3a,4,5,6,7,8,8b-octahydro-8,8-dimethyl
SKWC-24-1	Others	Butanoic acid, 2-methyl-, ethyl ester
	Linear hydrocarbons	Undecane
	Terpenoid	Linalool
	Terpenoid	.alpha.-Terpineol
	Linear hydrocarbons	Dodecane
	Others	4-Vinylphenol
	Terpenoid	Chavicol
	Others	2,4-Pentanedione, 3-(1-methyl-2-propenyl)-
	Others	Acetophenone, 4'-hydroxy-
	Others	1-Methyl-4-(6-methylhept-5-en-2-yl)cyclohexa-1,3-diene
	Phenylpropanoids	Benzoic acid, 2-methoxy-
	Terpenoid	.alpha.-Muurolene
	Terpenoid	.alpha.-Farnesene
	Terpenoid	1H-Benzocyclohepten-7-ol, 2,3,4,4a,5,6,7,8-octahydro-1,1,4a,7-tetramethyl-, cis-
	Terpenoid	Naphthalene, 1,2,3,4,4a,5,6,8a-octahydro-7-methyl-4-methylene-1-(1-methylethyl)-, (1.alpha.,4a.beta.,8a.alpha.)-
	Terpenoid	Naphthalene, 1,2,3,5,6,8a-hexahydro-4,7-dimethyl-1-(1-methylethyl)-, (1S-cis)-
	Phenylpropanoids	2-Butanone, 4-(4-hydroxyphenyl)-
	Terpenoid	1,6,10-Dodecatrien-3-ol, 3,7,11-trimethyl-, [S-(Z)]-
	Terpenoid	Farnesene epoxide, E-
	Terpenoid	.alpha.-Cadinol
	Phenylpropanoids	Benzene, 1,1'-[1-(2,2-dimethyl-3-butenyl)-1,3-propanediyl]bis-
	Phenylpropanoids	Benzene, 1,1'-(1,2-cyclobutanediyl)bis-, trans-
	Phenylpropanoids	Benzyl Benzoate
	Phenylpropanoids	Benzoic acid, 1-phenylethyl ester
	Phenylpropanoids	Benzoic acid, hept-2-yl ester
	Terpenoid	2-Cyclohexen-1-one, 2-methyl-5-(1-methylethenyl)-
	Phenylpropanoids	Benzoic acid, 2-hydroxy-, phenylmethyl ester
	Phenylpropanoids	2-Methoxybenzoic acid, benzyl ester
	Phenylpropanoids	2-Methoxybenzyl benzoate
	Phenylpropanoids	4-Methoxybenzyl benzoate
	Phenylpropanoids	(Z)-Cinnamyl benzoate
	Others	9,12-Octadecadienoic acid (Z,Z)-, methyl ester
	Phenylpropanoids	4-(Benzyloxy)-3-methoxybenzoic acid
	Linear hydrocarbons	Nonacosane
	Others	9-Octadecenamide, (Z)-
	Linear hydrocarbons	Tetracosane
	Phenylpropanoids	4H-1-Benzopyran-4-one, 2,3-dihydro-5-hydroxy-7-methoxy-2-phenyl-, (S)-
	Phenylpropanoids	Cinnamyl cinnamate
	Linear hydrocarbons	Tetratriacontane
	Phenylpropanoids	2',6'-Dihydroxy 4'-methoxydihydrochalcone, diacetate
	Phenylpropanoids	Benzenepropanoic acid, 3-phenyl-2-propenyl ester
	Phenylpropanoids	1,3,5-Triphenyl-1,5-pentanedione
	Linear hydrocarbons	Tricosane
	Phenylpropanoids	5-Hydroxy-4',7-dimethoxyflavanone
BESC-293	Others	Butanoic acid, 2-methyl-, ethyl ester
	Linear hydrocarbons	Dodecane, 2,6,11-trimethyl-
	Others	4-Vinylphenol
	Terpenoid	(-)-1,2,2.alpha.,3,3,4,6,7,8,8.alpha.-decahydro-2.alpha.,7,8-trimethylacenaphthylene
	Terpenoid	4,7-Methanoazulene, 1,2,3,4,5,6,7,8-octahydro-1,4,9,9-tetramethyl-
	Others	Acetophenone, 4'-hydroxy-
	Terpenoid	1,6,10-Dodecatrien-3-ol, 3,7,11-trimethyl-, [S-(Z)]-
	Terpenoid	2-Adamantanol, 2-(bromomethyl)-
	Terpenoid	.tau.-Cadinol
	Phenylpropanoids	Benzene, 1,1'-(1,2-cyclobutanediyl)bis-, trans-
	Phenylpropanoids	Benzyl Benzoate
	Phenylpropanoids	Benzoic acid, hept-2-yl ester
	Terpenoid	2-Cyclohexen-1-one, 2-methyl-5-(1-methylethenyl)-
	Phenylpropanoids	Benzoic acid, 2-hydroxy-, phenylmethyl ester
	Phenylpropanoids	2-Methoxybenzoic acid, benzyl ester
	Terpenoid	1(2H)-Naphthalenone, 3,4-dihydro-4-phenyl-
	Others	Isopropyl palmitate
	Phenylpropanoids	(Z)-Cinnamyl benzoate
	Linear hydrocarbons	Tetracosane
	Others	9-Octadecenamide, (Z)-
	Phenylpropanoids	4H-1-Benzopyran-4-one, 2,3-dihydro-5-hydroxy-7-methoxy-2-phenyl-, (S)-
	Phenylpropanoids	Cinnamyl cinnamate
	Terpenoid	4H-1-Benzopyran-4-one, 2,3-dihydro-5,7-dihydroxy-2-phenyl-, (S)-
	Phenylpropanoids	4H-1-Benzopyran-4-one, 5-hydroxy-7-methoxy-2-phenyl-
	Phenylpropanoids	2',6'-Dihydroxy 4'-methoxydihydrochalcone, diacetate
	Others	4H-1-Benzopyran-4-one, 5,7-dihydroxy-2-phenyl-
	Phenylpropanoids	5-Hydroxy-4',7-dimethoxyflavanone
	Phenylpropanoids	Isosakuranetin, diacetate

	Terpenoids	2-Pinene
	Linear hydrocarbons	Decane
	Linear hydrocarbons	Undecane
	Terpenoids	Linalool
	Terpenoids	Eucalyptol
	Terpenoids	2-Oxabicyclo[2.2.2]octane, 1,3,3-trimethyl-
	Terpenoids	Estragole
	Terpenoids	Beta-cyclocitral
	Linear hydrocarbons	Tetradecane
	Linear hydrocarbons	Dodecane, 2-methyl-
	Terpenoids	4,7-Methanoazulene, 1,2,3,4,5,6,7,8-octahydro-1,4,9,9-tetramethyl-
	Terpenoids	1H-Cycloprop[e]azulene, decahydro-1,1,7-trimethyl-4-methylene-, (1aR,4aS,7R,7aR,7bS)-(-)-
	Terpenoids	1,2,4-Metheno-1H-indene, octahydro-1,7a-dimethyl-5-(1-methylethyl)-, [1S-(1.alpha.,2.alpha.,3a.beta.,4.alpha.,5.alpha.,7a.beta.
	Terpenoids	Naphthalene, decahydro-4a-methyl-1-methylene-7-(1-methylethenyl)-, [4aR-(4a.alpha.,7.alpha.,8a.beta.)]-
	Terpenoids	4.beta.H,5.alpha.-Eremophila-1(10),11-diene
	Terpenoids	1H-Benzocyclohepten-7-ol, 2,3,4,4a,5,6,7,8-octahydro-1,1,4a,7-tetramethyl-, cis-
	Terpenoids	trans-.alpha.-Bergamotene
	Others	Phenol, 2,4-bis(1,1-dimethylethyl)-
	Terpenoids	Zonarene
	Terpenoids	1,6,10-Dodecatrien-3-ol, 3,7,11-trimethyl-, [S-(Z)]-
	Terpenoids	1,6,10-Dodecatrien-3-ol, 3,7,11-trimethyl-, (E)-
	Terpenoids	2-(4a,8-Dimethyl-2,3,4,5,6,7-hexahydro-1H-naphthalen-2-yl)propan-2-ol
	Terpenoids	Isoaromadendrene epoxide
	Terpenoids	2-Naphthalenemethanol, 1,2,3,4,4a,5,6,8a-octahydro-.alpha.,.alpha.,4a,8-tetramethyl-, [2R-(2.alpha.,4a.alpha.,8a.beta.)]-
	Terpenoids	3-Cyclohexene-1-methanol, .alpha.,4-dimethyl-.alpha.-(4-methyl-3-penten
	Terpenoids	2,6,10-Dodecatrien-1-ol, 3,7,11-trimethyl-
BESC-1015	Linear hydrocarbons	1-Octadecene
	Phenylpropanoids	3-Hexen-1-ol benzoate
	Terpenoids	[(1S,7S,8S,8aS)-8-[2-[(2R,4R)-4-Hydroxy-6-oxooxan-2-yl]ethyl]-7-methyl-1,2,3,4,4a,7,8,8a-octahydronaphthalen-1-yl] (2S)-2-methy
	Terpenoids	Neophytadiene
	Terpenoids	Boscartol F
	Terpenoids	1(2H)-Naphthalenone, 3,4-dihydro-4-phenyl-
	Terpenoids	alpha-sinesal
	Others	Isoheptadecanol
	Linear hydrocarbons	1-Tricosene
	Phenylpropanoids	4-Methoxybenzyl benzoate
	Phenylpropanoids	Benzoic acid, 2-methoxy-, methyl ester
	Phenylpropanoids	piceol
	Linear hydrocarbons	Tetracosane
	Linear hydrocarbons	Hexacosane
	Phenylpropanoids	(-)-Pinostrobin
	Phenylpropanoids	2-Butanone, 4-(4-hydroxyphenyl)-
	Phenylpropanoids	Pinostrobin chalcone
	Phenylpropanoids	Benzyl trans-4-coumarate
	Linear hydrocarbons	Nonacosane
	Linear hydrocarbons	Tricosane
	Others	1-Heptacosanol
	Phenylpropanoids	(E)-hinokiresinol
	Terpenoids	Naphthalene, 1,2,3,4-tetrahydro-1-phenyl-
	Linear hydrocarbons	Tetratriacontane
	Linear hydrocarbons	Triacontane, 1-bromo-
	Linear hydrocarbons	Pentatriacontane
	Others	Cholesterol
	Others	1,6,10-Dodecatrien-3-ol, 3,7,11-trimethyl-, [S-(Z)]-
	Others	Butanoic acid, 3-methyl-, ethyl ester
	Linear hydrocarbons	2-Butanone
	Linear hydrocarbons	Nonane, 2,5-dimethyl-
	Linear hydrocarbons	Dodecane
	Phenylpropanoids	Dodecane, 2,6,11-trimethyl-
	Phenylpropanoids	Benzoic acid, 1-methylpropyl ester
	Phenylpropanoids	2-Propen-1-ol, 3-phenyl-
	Phenylpropanoids	trans-Cinnamic acid
	Terpenoids	1-Ethyl-4-methoxybenzene
	Terpenoids	Naphthalene, decahydro-4a-methyl-1-methylene-7-(1-methylethenyl)-, [4aR-(4a.alpha.,7.alpha.,8a.beta.)]-
	Terpenoids	zerumbone
	Linear hydrocarbons	1-Octadecanesulphonyl chloride
	Terpenoids	4,11-Dimethyl-8-(propan-2-yl)-5,12-dioxatricyclo[9.1.0.04,6]dodecan-7-ol, Ac
	Terpenoids	4a(2H)-Naphthalenol, 1,3,4,5,6,8a-hexahydro-4,7-dimethyl-1-(1-methylethyl)-, (1S,4S,4aS,8aR)-
	Phenylpropanoids	Phenol, 2,4-di-t-butyl-6-nitro-
	Terpenoids	3-Cyclohexene-1-methanol, .alpha.,4-dimethyl-.alpha.-(4-methyl-3-penten
	Phenylpropanoids	2-Hexanone, 6-phenyl-
	Phenylpropanoids	4-Pentenoic acid, 5-phenyl-
	Phenylpropanoids	Benzene, 1,1'-(1,2-cyclobutanediyl)bis-, trans-
BESC-109	Terpenoids	(-)-Spathulenol
	Phenylpropanoids	Benzoic acid, 1-phenylethyl ester
	Phenylpropanoids	3-Hexen-1-ol benzoate
	Phenylpropanoids	Benzoic acid, 2-hydroxy-, phenylmethyl ester
	Linear hydrocarbons	Isoheptadecanol
	Linear hydrocarbons	1-Tricosene
	Phenylpropanoids	2-Methoxybenzyl benzoate
	Phenylpropanoids	2-Phenylpropionsaeure
	Phenylpropanoids	Benzeneacetic acid, methyl ester
	Linear hydrocarbons	Octadecanoic acid
	Phenylpropanoids	4-(Benzyloxy)-2-nitroaniline
	Linear hydrocarbons	Hexacosane
	Phenylpropanoids	2-Propen-1-one, 1-(2,6-dihydroxy-4-methoxyphenyl)-3-phenyl-, (E)-
	Phenylpropanoids	2-Butanone, 4-(4-hydroxyphenyl)-
	Phenylpropanoids	Benzyl trans-4-coumarate
	Linear hydrocarbons	2-Methylhexacosane
	Phenylpropanoids	8-Hydroxy-2,2,5-trimethyl-5-(3-methylbut-2-en-1-yl)-7-(3-phenylpropanoyl)-2H-chromen-6(5H)-one

	Phenylpropanoids	2,2,5-trimethyl-5-(3-methylbut-2-enyl)-8-oxidanyl-7-(3-phenylpropanoyl)chromen-6-one
	Phenylpropanoids	4H-1-Benzopyran-4-one, 2,3-dihydro-5-hydroxy-2-(4-hydroxyphenyl)-7-methoxy-, (S)-
	Others	14,16-Hentriacontanedione
	Linear hydrocarbons	Pentatriacontane
BESC-110	Linear hydrocarbons	Decane
	Others	2-Pyrrolidinone, 1-(2-aminoethyl)-
	Linear hydrocarbons	Nonane, 2,5-dimethyl-
	Linear hydrocarbons	Undecane
	Terpenoids	Linalool
	Terpenoids	(1S)-1,3,3-trimethylnorbornan-2-ol
	Terpenoids	Estragole
	Others	Nonanal
	Linear hydrocarbons	Dodecane
	Phenylpropanoids	Benzoic acid
	Phenylpropanoids	Benzoic acid, 1-methylpropyl ester
	Linear hydrocarbons	Tetradecane
	Linear hydrocarbons	Dodecane, 2-methyl-
	Linear hydrocarbons	Hexadecane
	Phenylpropanoids	2-Propenoic acid, 3-phenyl-
	Terpenoids	4,7-Methanoazulene, 1,2,3,4,5,6,7,8-octahydro-1,4,9,9-tetramethyl-
	Phenylpropanoids	Acetophenone, 4'-hydroxy-
	Phenylpropanoids	Ethanone, 1-(2-hydroxyphenyl)-
	Phenylpropanoids	Ethanone, 1-(3-hydroxyphenyl)-
	Terpenoids	1H-Cycloprop[e]azulene, decahydro-1,1,7-trimethyl-4-methylene-, (1aR,4aS,7R,7aR,7bS)-(-)-
	Terpenoids	(1R,4aS,8aR)-1-Isopropyl-4,7-dimethyl-1,2,4a,5,6,8a-hexahydronaphthalene
	Phenylpropanoids	Benzoic acid, 2-methoxy-
	Terpenoids	Copaene
	Others	1-{3-tricyclo[2.2.1.0(2.6)]heptyl}-1-butanone
	Linear hydrocarbons	1-Octadecanesulphonyl chloride
	Terpenoids	Farnesene epoxide, E-
	Terpenoids	Hinesol
	Terpenoids	Agarospirol
	Phenylpropanoids	2-Hexanone, 6-phenyl-
	Phenylpropanoids	Benzene, 1,1'-[1-(2,2-dimethyl-3-butenyl)-1,3-propanediyl]bis-
	Phenylpropanoids	4-Pentenoic acid, 5-phenyl-
	Others	Bromoacetic acid, pentadecyl ester
	Phenylpropanoids	Benzoic acid, 1-phenylethyl ester
	Linear hydrocarbons	Octadecane, 1-chloro-
	Phenylpropanoids	Benzoic acid, hept-2-yl ester
	Phenylpropanoids	3-Hexen-1-ol benzoate
	Terpenoids	1(2H)-Naphthalenone, 3,4-dihydro-4-phenyl-
	Terpenoids	(2E,6E,9E)-2,6,10-Trimethyl-2,6,9,11-dodecatetraenal
	Others	Hexadecanamide, N-(2-hydroxyethyl)-
BESC-1120	Linear hydrocarbons	Isoheptadecanol
	Linear hydrocarbons	1-Tricosene
	Others	Name
	Phenylpropanoids	Benzene, [[(1-ethenyl-1,5-dimethyl-4-hexenyl)oxy]methyl]-
	Phenylpropanoids	4-Methoxybenzyl benzoate
	Phenylpropanoids	Benzoic acid, 2-methoxy-, methyl ester
	Phenylpropanoids	2-Methoxybenzoic acid, 2-phenylethyl ester
	Phenylpropanoids	3-Phenylpropionyl fluoride-2,2-D2
	Phenylpropanoids	Benzoic acid, 2-hydroxy-4-[(6-hydroxy-3,4-dimethoxy-2-methylbenzoyl)oxy]-6-methyl-, 3-hydroxy-5-methyl-4-[(phenylmethoxy)carbon
	Phenylpropanoids	4-(Benzyloxy)-3-methoxybenzoic acid
	Linear hydrocarbons	Octadecanoic acid
	Phenylpropanoids	1-Butanone, 1,4-diphenyl-
	Linear hydrocarbons	Eicosane
	Linear hydrocarbons	N-heneicosane
	Linear hydrocarbons	Hexacosane
	Linear hydrocarbons	Tetracosane
	Phenylpropanoids	Pinostrobin chalcone
	Phenylpropanoids	Benzyl trans-4-coumarate
	Linear hydrocarbons	2-Methylhexacosane
	Phenylpropanoids	4H-1-Benzopyran-4-one, 5-hydroxy-7-methoxy-2-phenyl-
	Phenylpropanoids	2',6'-Dihydroxy 4'-methoxydihydrochalcone, diacetate
	Phenylpropanoids	2',4',6'-Trihydroxydihydrochalcone
	Linear hydrocarbons	Tricosane
	Phenylpropanoids	2,2,5-trimethyl-5-(3-methylbut-2-enyl)-8-oxidanyl-7-(3-phenylpropanoyl)chromen-6-one
	Others	1-Heptacosanol
	Phenylpropanoids	5-Hydroxy-4',7-dimethoxyflavanone
	Others	(E)-hinokiresinol
	Phenylpropanoids	Naphthalene, 1,2,3,4-tetrahydro-1-phenyl-
	Phenylpropanoids	Galangin
	Others	Ethanone, 1-[2,3-dihydro-6-hydroxy-2-(1-hydroxy-1-methylethyl)-4-methoxy-7-benzofuranyl]-, (+)-
	Phenylpropanoids	Isosakuranetin, diacetate
	Others	Hexanoic acid, heptadecyl ester
	Linear hydrocarbons	Tetratriacontane
	Linear hydrocarbons	Triacontane, 1-bromo-
	Phenylpropanoids	Benzoic acid, [(E,E)-3,7,11-trimethyl-2,6,10-dodecatrien-1-yl] ester
	Others	2-Hexyldodecyl acetate
	Linear hydrocarbons	Pentatriacontane
	Others	Eicosyl nonyl ether
	Others	2H-Indeno[1,2-b]furan-2-one, 3,3a,4,5,6,7,8,8b-octahydro-8,8-dimethyl
	Others	cis-13-Eicosenoic acid
	Others	(Tetrahydro-2H-pyran-2-yl)methyl 4-methylpentanoate
	Terpenoids	Beta-cyclocitral
	Others	4-Vinylphenol
	Phenylpropanoids	trans-Cinnamic acid
	Phenylpropanoids	Benzoic acid, 2-methoxy-
	Terpenoids	1,6,10-Dodecatrien-3-ol, 3,7,11-trimethyl-, (E)-
	Phenylpropanoids	Phenol, 2,4-di-t-butyl-6-nitro-

	Phenylpropanoids	Benzoic acid, 1-phenylethyl ester
	Phenylpropanoids	Phenylethyl salicylate
	Phenylpropanoids	4-Methoxybenzyl benzoate
	Phenylpropanoids	(E)-Cinnamyl benzoate
	Phenylpropanoids	2-Methoxybenzoic acid, 2-phenylethyl ester
BESC-1174	Phenylpropanoids	2-Phenylpropionsaeure
	Terpenoids	1.6-Farnesadiene-3.10.11-triol, acetate
	Linear hydrocarbons	Octadecanoic acid
	Linear hydrocarbons	Hexacosane
	Linear hydrocarbons	Tetracosane
	Phenylpropanoids	(-)-Pinostrobin
	Phenylpropanoids	4H-1-Benzopyran-4-one, 2,3-dihydro-5-hydroxy-7-methoxy-2-phenyl-, (S)-
	Linear hydrocarbons	2-Methylhexacosane
	Linear hydrocarbons	Nonacosane
	Linear hydrocarbons	Tricosane
	Phenylpropanoids	(E)-hinokiresinol
	Linear hydrocarbons	Nonane, 2,5-dimethyl-
	Linear hydrocarbons	Undecane
	Terpenoids	2-Oxabicyclo[2.2.2]octane, 1,3,3-trimethyl-
	Terpenoids	Estragole
	Terpenoids	4-terpineol
	Linear hydrocarbons	Dodecane
	Linear hydrocarbons	Dodecane, 2,6,11-trimethyl-
	Phenylpropanoids	Benzoic acid, 1-methylpropyl ester
	Linear hydrocarbons	Dodecane, 2-methyl-
	Linear hydrocarbons	Tetradecane
	Terpenoids	4,7-Methanoazulene, 1,2,3,4,5,6,7,8-octahydro-1,4,9,9-tetramethyl-
	Phenylpropanoids	Acetophenone, 4'-hydroxy-
	Phenylpropanoids	trans-Cinnamic acid
	Phenylpropanoids	Ethanone, 1-(2-hydroxyphenyl)-
	Phenylpropanoids	1-Ethyl-4-methoxybenzene
	Phenylpropanoids	(p-Hydroxyphenyl)glyoxal
	Phenylpropanoids	Ethanone, 1-(3-hydroxyphenyl)-
	Terpenoids	1H-Cycloprop[e]azulene, decahydro-1,1,7-trimethyl-4-methylene-, (1aR,4aS,7R,7aR,7bS)-(-)-
	Terpenoids	.gamma.-Muurolene
	Terpenoids	Italicene
	Terpenoids	Naphthalene, 1,2,3,4,4a,5,6,8a-octahydro-7-methyl-4-methylene-1-(1-methylethyl)-, (1.alpha.,4a.beta.,8a.alpha.)-
	Terpenoids	Zonarene
	Linear hydrocarbons	1-Octadecanesulphonyl chloride
	Phenylpropanoids	Phenol, 2,4-di-t-butyl-6-nitro-
	Terpenoids	3-Cyclohexene-1-methanol, .alpha.,4-dimethyl-.alpha.-(4-methyl-3-penten
	Phenylpropanoids	2-Hexanone, 6-phenyl-
	Phenylpropanoids	4-Pentenoic acid, 5-phenyl-
	Linear hydrocarbons	1-Octadecene
	Phenylpropanoids	Benzoic acid, 1-phenylethyl ester
	Linear hydrocarbons	Octadecane, 1-chloro-
	Phenylpropanoids	Benzoic acid, hept-2-yl ester
BESC-1186	Terpenoids	alpha-sinesal
	Linear hydrocarbons	1-Tricosene
	Phenylpropanoids	Benzene, [[(1-ethenyl-1,5-dimethyl-4-hexenyl)oxy]methyl]-
	Phenylpropanoids	4-Methoxybenzyl benzoate
	Phenylpropanoids	(E)-Cinnamyl benzoate
	Others	9,12-Octadecadienoic acid (Z,Z)-, methyl ester
	Phenylpropanoids	Benzoic acid, 2-methoxy-, methyl ester
	Phenylpropanoids	2-Phenylpropionsaeure
	Phenylpropanoids	3-Phenylpropionyl fluoride-2,2-D2
	Phenylpropanoids	Benzeneacetic acid, methyl ester
	Phenylpropanoids	4-(Benzyloxy)-3-methoxybenzoic acid
	Phenylpropanoids	piceol
	Linear hydrocarbons	Octadecanoic acid
	Others	Acetic acid n-octadecyl ester
	Phenylpropanoids	4-(Benzyloxy)-2-nitroaniline
	Phenylpropanoids	1-Butanone, 1,4-diphenyl-
	Linear hydrocarbons	Eicosane
	Phenylpropanoids	(-)-Pinostrobin
	Phenylpropanoids	Pinostrobin chalcone
	Linear hydrocarbons	2-Methylhexacosane
	Linear hydrocarbons	Hexatriacontane
	Phenylpropanoids	2',6'-Dihydroxy 4'-methoxydihydrochalcone, diacetate
	Phenylpropanoids	Benzofuran-6-ol-3-one, 2-(4-ethoxycarbonyl)benzylidene-
	Linear hydrocarbons	Nonacosane
	Linear hydrocarbons	Tricosane
	Others	1-Heptacosanol
	Phenylpropanoids	5-Hydroxy-4',7-dimethoxyflavanone
	Phenylpropanoids	(E)-hinokiresinol
	Phenylpropanoids	Naphthalene, 1,2,3,4-tetrahydro-1-phenyl-
	Phenylpropanoids	Benzoic acid, [(E,E)-3,7,11-trimethyl-2,6,10-dodecatrien-1-yl] ester
	Linear hydrocarbons	Pentatriacontane
	Others	cis-13-Eicosenoic acid
	Terpenoids	(-)-alpha-fenchol
	Terpenoids	beta-cyclocitral
	Terpenoids	1-Cyclohexene-1-carboxaldehyde, 2,6,6-trimethyl-
	Others	Acetophenone, 4'-hydroxy-
	Terpenoids	(1R,3aS,4aS,8aS)-1,4,4,6-Tetramethyl-1,2,3,3a,4,4a,7,8-octahydrocyclopenta[1,4]cyclobuta[1,2]benzene
	Terpenoids	1,2,4-Metheno-1H-indene, octahydro-1,7a-dimethyl-5-(1-methylethyl)-, [1S-(1.alpha.,2.alpha.,3a.beta.,4.alpha.,5.alpha.,7a.beta.
	Terpenoids	Naphthalene, 1,2,4a,5,6,8a-hexahydro-4,7-dimethyl-1-(1-methylethyl)-
	Terpenoids	4a(2H)-Naphthalenol, 1,3,4,5,6,8a-hexahydro-4,7-dimethyl-1-(1-methylethyl)-, (1S,4S,4aS,8aR)-
	Terpenoids	Hinesol
	Terpenoids	2-Naphthalenemethanol, decahydro-.alpha.,.alpha.,4a-trimethyl-8-methylene-, [2R-(2.alpha.,4a.alpha.,8a.beta.)]-
	Terpenoids	Isoaromadendrene epoxide

BESC-12	Phenylpropanoids	[3,3-Dimethyl-1-(2-phenylethyl)-4-pentenyl]benzene
	Linear hydrocarbons	1-Octadecene
	Terpenoids	[(1S,7S,8S,8aS)-8-[2-[(2R,4R)-4-Hydroxy-6-oxooxan-2-yl]ethyl]-7-methyl-1,2,3,4,4a,7,8,8a-octahydronaphthalen-1-yl] (2S)-2-methy
	Terpenoids	1(2H)-Naphthalenone, 3,4-dihydro-4-phenyl-
	Terpenoids	alpha-sinesal
	Phenylpropanoids	(Z)-Cinnamyl benzoate
	Phenylpropanoids	3-Phenylpropionyl fluoride-2,2-D2
	Phenylpropanoids	4-(Benzyloxy)-3-methoxybenzoic acid
	Others	Benzamide, N-(2-cyanocyclopent-1-enyl)-3,4-dimethoxy-
	Linear hydrocarbons	Tetracosane
	Linear hydrocarbons	2-Methylhexacosane
	Linear hydrocarbons	Triacontane, 1-bromo-
	Terpenoids	Squalene
BESC-389	Others	Butanoic acid, 3-methyl-, ethyl ester
	Others	Butanoic acid, 2-methyl-, ethyl ester
	Others	2-Butanone
	Linear hydrocarbons	Nonane, 2,5-dimethyl-
	Linear hydrocarbons	Undecane
	Others	Nonanal
	Linear hydrocarbons	Dodecane, 2-methyl-
	Linear hydrocarbons	Tetradecane
	Terpenoids	(1R,3aS,4aS,8aS)-1,4,4,6-Tetramethyl-1,2,3,3a,4,4a,7,8-octahydrocyclopenta[1,4]cyclobuta[1,2]benzene
	Others	1,6-Dimethyl-4-propan-2-yl-1,2,3,7,8,8a-hexahydronaphthalene
	Terpenoids	2-Naphthalenemethanol, decahydro-.alpha.,.alpha.,4a-trimethyl-8-methylene-, [2R-(2.alpha.,4a.alpha.,8a.beta.)]-
	Others	Phenol, 2,4-di-t-butyl-6-nitro-
	Phenylpropanoids	[3,3-Dimethyl-1-(2-phenylethyl)-4-pentenyl]benzene
	Terpenoids	(-)-Spathulenol
	Terpenoids	4-(2,2,6-Trimethylcyclohexyl)-2-butanone
	Linear hydrocarbons	Isoheptadecanol
	Others	1-Hexadecanol, 2-methyl-
	Others	1-Hexadecanol
	Others	1-Nonadecene
	Phenylpropanoids	3-Phenylpropionyl fluoride-2,2-D2
	Phenylpropanoids	2-Phenylpropionsaeure
	Others	2,4-Pentanedione, 3-(1-methyl-2-propenyl)-
	Linear hydrocarbons	Octadecanoic acid
	Others	2-Oxazolamine, 4,5-dihydro-5-(phenoxymethyl)-N-[(phenylamino)carbonyl]-
	Linear hydrocarbons	Eicosane
	Phenylpropanoids	Pinostrobin chalcone
	Phenylpropanoids	Benzyl trans-4-coumarate
	Linear hydrocarbons	2-Methylhexacosane
	Linear hydrocarbons	Hexatriacontane
	Phenylpropanoids	4H-1-Benzopyran-4-one, 5-hydroxy-7-methoxy-2-phenyl-
	Others	2',6'-Dihydroxy 4'-methoxydihydrochalcone, diacetate
	Others	2',4',6'-Trihydroxydihydrochalcone
	Phenylpropanoids	Benzenepropanoic acid, 3-phenyl-2-propenyl ester
	Linear hydrocarbons	Nonacosane
	Phenylpropanoids	2,2,5-trimethyl-5-(3-methylbut-2-enyl)-8-oxidanyl-7-(3-phenylpropanoyl)chromen-6-one
	Others	norlignan
	Phenylpropanoids	4H-1-Benzopyran-4-one, 2,3-dihydro-5-hydroxy-2-(4-hydroxyphenyl)-7-methoxy-, (S)-
	Linear hydrocarbons	Tetratriacontane
	Linear hydrocarbons	Triacontane, 1-bromo-
	Others	2-Hexyldodecyl acetate
BESC-853	Others	Butanoic acid, 3-methyl-, ethyl ester
	Linear hydrocarbons	Decane
	Others	D-Limonene
	Linear hydrocarbons	Nonane, 2,5-dimethyl-
	Linear hydrocarbons	Undecane
	Terpenoids	Eucalyptol
	Terpenoids	Estragole
	Linear hydrocarbons	Dodecane, 2,6,11-trimethyl-
	Linear hydrocarbons	Dodecane, 2-methyl-
	Phenylpropanoids	Acetophenone, 4'-hydroxy-
	Phenylpropanoids	Ethanone, 1-(3-hydroxyphenyl)-
	Terpenoids	trans-Z-.alpha.-Bisabolene epoxide
	Terpenoids	3-Cyclohexene-1-methanol, .alpha.,4-dimethyl-.alpha.-(4-methyl-3-penten
	Phenylpropanoids	[3,3-Dimethyl-1-(2-phenylethyl)-4-pentenyl]benzene
	Phenylpropanoids	Benzene, 1,1'-[1-(2,2-dimethyl-3-butenyl)-1,3-propanediyl]bis-
	Others	3-Methyl-2-butenoic acid, tridec-2-ynyl ester
	Terpenoids	2,6,10-Dodecatrien-1-ol, 3,7,11-trimethyl-
	Others	Tetradecanoic acid
	Linear hydrocarbons	Isoheptadecanol
	Others	1-Hexadecanol, 2-methyl-
	Others	1-Nonadecene
	Others	Hexadecanoic acid, methyl ester
	Phenylpropanoids	4-Methoxybenzyl benzoate
	Others	Isopropyl palmitate
	Phenylpropanoids	3-Phenylpropionyl fluoride-2,2-D2
	Others	11(Z),14(Z),17(Z)-Eicosatrienoic Acid methyl ester
	Linear hydrocarbons	Octadecanoic acid
	Phenylpropanoids	4-(Benzyloxy)-2-nitroaniline
	Others	2-Oxazolamine, 4,5-dihydro-5-(phenoxymethyl)-N-[(phenylamino)carbonyl]-
	Phenylpropanoids	1,3,5-Triphenyl-1,5-pentanedione
	Others	N,N-dimethylhexadecanamide
	Linear hydrocarbons	Eicosane
	Linear hydrocarbons	N-heneicosane
	Linear hydrocarbons	Hexacosane
	Linear hydrocarbons	Tetracosane
	Phenylpropanoids	Benzyl trans-4-coumarate
	Linear hydrocarbons	Nonacosane

	Linear hydrocarbons	Tricosane
	Others	(E)-hinokiresinol
	Phenylpropanoids	Naphthalene, 1,2,3,4-tetrahydro-1-phenyl-
	Others	Ethanone, 1-[2,3-dihydro-6-hydroxy-2-(1-hydroxy-1-methylethyl)-4-methoxy-7-benzofuranyl]-, (+)-
	Linear hydrocarbons	Tetratriacontane
	Linear hydrocarbons	Triacontane, 1-bromo-
	Others	14,16-Hentriacontanedione
	Others	Eicosyl nonyl ether
	Others	2H-Indeno[1,2-b]furan-2-one, 3,3a,4,5,6,7,8,8b-octahydro-8,8-dimethyl
BESC-904	Others	Acetic acid, butyl ester
	Others	Butanoic acid, 2-methyl-, ethyl ester
	Linear hydrocarbons	Decane
	Terpenoids	Linalool
	Terpenoids	2-Oxabicyclo[2.2.2]octane, 1,3,3-trimethyl-
	Linear hydrocarbons	Dodecane
	Linear hydrocarbons	Dodecane, 2,6,11-trimethyl-
	Others	1-Cyclohexene-1-carboxaldehyde, 2,6,6-trimethyl-
	Terpenoids	Chavicol
	Phenylpropanoids	Benzoic acid, 1-methylpropyl ester
	Others	Benzaldehyde, 4-hydroxy-
	Linear hydrocarbons	Tetradecane
	Linear hydrocarbons	Dodecane, 2-methyl-
	Terpenoids	Naphthalene, decahydro-4a-methyl-1-methylene-7-(1-methylethenyl)-, [4aR-(4a.alpha.,7.alpha.,8a.beta.)]-
	Others	Phenol, 2,4-bis(1,1-dimethylethyl)-
	Linear hydrocarbons	1-Octadecanesulphonyl chloride
	Linear hydrocarbons	Octadecane, 1-chloro-
	Terpenoids	Boscartol F
	Phenylpropanoids	Benzoic acid, 2-hydroxy-, phenylmethyl ester
	Others	Isoheptadecanol
	Others	1-Hexadecanol
	Linear hydrocarbons	1-Tricosene
	Others	n-Hexadecanoic acid
	Phenylpropanoids	2-Methoxybenzoic acid, benzyl ester
	Phenylpropanoids	(E)-Cinnamyl benzoate
	Others	2,4-Pentanedione, 3-(1-methyl-2-propenyl)-
	Phenylpropanoids	Benzoic acid, 2-hydroxy-4-[(6-hydroxy-3,4-dimethoxy-2-methylbenzoyl)oxy]-6-methyl-, 3-hydroxy-5-methyl-4-[(phenylmethoxy)carbon
	Phenylpropanoids	Benzeneacetic acid, methyl ester
	Phenylpropanoids	piceol
	Others	Name
	Others	N,N-dimethylhexadecanamide
	Others	9-Octadecenamide
	Linear hydrocarbons	Tetracosane
	Linear hydrocarbons	2-Methylhexacosane
	Linear hydrocarbons	Hexatriacontane
	Phenylpropanoids	4H-1-Benzopyran-4-one, 5-hydroxy-7-methoxy-2-phenyl-
	Others	4H-1-Benzopyran-4-one, 5,7-dihydroxy-2-phenyl-
	Linear hydrocarbons	Tricosane
	Phenylpropanoids	Sakuranetin
	Others	Isosakuranetin, diacetate
	Linear hydrocarbons	Tetratriacontane
	Linear hydrocarbons	Triacontane, 1-bromo-
	Linear hydrocarbons	Pentatriacontane
BESC-29	Others	Acetic acid ethyl ester
	Others	2,4-Dimethyl-1-heptene
	Linear hydrocarbons	Undecane
	Linear hydrocarbons	Dodecane
	Linear hydrocarbons	Dodecane, 2,6,11-trimethyl-
	Others	Name
	Phenylpropanoids	4-Vinylphenol
	Linear hydrocarbons	Dodecane, 2-methyl-
	Linear hydrocarbons	Hexadecane
	Others	1,6-Dimethyl-4-propan-2-yl-1,2,3,7,8,8a-hexahydronaphthalene
	Terpenoids	trans-.alpha.-Bergamotene
	Terpenoids	(-)-1,2,2.alpha.,3,3,4,6,7,8,8.alpha.-decahydro-2.alpha.,7,8-trimethylacenaphthylene
	Terpenoids	gamma-ionone
	Terpenoids	Isoaromadendrene epoxide
	Phenylpropanoids	Phenol, 2,4-di-t-butyl-6-nitro-
	Phenylpropanoids	Benzene, 1,1'-(1,2-cyclobutanediyl)bis-, trans-
	Linear hydrocarbons	Octadecane, 1-chloro-
	Terpenoids	(2E,6E,9E)-2,6,10-Trimethyl-2,6,9,11-dodecatetraenal
	Others	2-(2,2-Dimethylpropanoyl)cyclohexanone
	Linear hydrocarbons	Isoheptadecanol
	Others	1-Hexadecanol
	Linear hydrocarbons	1-Tricosene
	Phenylpropanoids	(Z)-Cinnamyl benzoate
	Others	9,12-Octadecadienoic acid (Z,Z)-, methyl ester
	Phenylpropanoids	3-Phenylpropionyl fluoride-2,2-D2
	Phenylpropanoids	Benzoic acid, 2-hydroxy-4-[(6-hydroxy-3,4-dimethoxy-2-methylbenzoyl)oxy]-6-methyl-, 3-hydroxy-5-methyl-4-[(phenylmethoxy)carbon
	Phenylpropanoids	Benzeneacetic acid, methyl ester
	Linear hydrocarbons	Octadecanoic acid
	Others	N,N-dimethylhexadecanamide
	Linear hydrocarbons	Eicosane
	Others	9-Octadecenamide, (Z)-
	Linear hydrocarbons	N-heneicosane
	Linear hydrocarbons	Tetracosane
	Phenylpropanoids	pinostrobin chalcone
	Phenylpropanoids	Cinnamyl cinnamate
	Linear hydrocarbons	Hexatriacontane
	Others	4H-1-Benzopyran-4-one, 5,7-dihydroxy-2-phenyl-
	Others	5-Hydroxy-4',7-dimethoxyflavanone

	Linear hydrocarbons	Tetratriacontane
	Linear hydrocarbons	Triacontane, 1-bromo-
	Others	Pentatriacontane
	Others	2H-Indeno[1,2-b]furan-2-one, 3,3a,4,5,6,7,8,8b-octahydro-8,8-dimethyl
	Others	Cholesterol
	Terpenoids	Eucalyptol
	Phenylpropanoids	Benzoic acid
	Linear hydrocarbons	Tetradecane
	Linear hydrocarbons	Dodecane, 2-methyl-
	Linear hydrocarbons	Hexadecane
	Phenylpropanoids	Acetophenone, 4'-hydroxy-
	Terpenoids	(1R,3aS,4aS,8aS)-1,4,4,6-Tetramethyl-1,2,3,3a,4,4a,7,8-octahydrocyclopenta[1,4]cyclobuta[1,2]benzene
	Terpenoids	1,2,4-Metheno-1H-indene, octahydro-1,7a-dimethyl-5-(1-methylethyl)-, [1S-(1.alpha.,2.alpha.,3a.beta.,4.alpha.,5.alpha.,7a.beta.
	Phenylpropanoids	Benzoic acid, 2-methoxy-
	Terpenoids	Copaene
	Terpenoids	trans-L-Carvyl isobutyrate
	Terpenoids	2,6,10-Dodecatrien-1-ol, 3,7,11-trimethyl-
	Phenylpropanoids	Benzene, 1,1'-(1,2-cyclobutanediyl)bis-, trans-
	Linear hydrocarbons	Octadecane, 1-chloro-
	Phenylpropanoids	3-Hexen-1-ol benzoate
	Phenylpropanoids	Benzoic acid, 2-hydroxy-, phenylmethyl ester
	Phenylpropanoids	2-Methoxybenzoic acid, benzyl ester
	Phenylpropanoids	(E)-Cinnamyl benzoate
DENA-17-1	Phenylpropanoids	2-Methoxybenzoic acid, 2-phenylethyl ester
	Phenylpropanoids	2-Phenylpropionsaeure
	Phenylpropanoids	Benzoic acid, 2-hydroxy-4-[(6-hydroxy-3,4-dimethoxy-2-methylbenzoyl)oxy]-6-methyl-, 3-hydroxy-5-methyl-4-[(phenylmethoxy)carbon
	Phenylpropanoids	Benzeneacetic acid, methyl ester
	Phenylpropanoids	4-(Benzyloxy)-3-methoxybenzoic acid
	Others	Benzamide, N-(2-cyanocyclopent-1-enyl)-3,4-dimethoxy-
	Phenylpropanoids	Benzenemethanol, 2-hydroxy-3,6-dimethyl-4-(phenylmethoxy)-
	Phenylpropanoids	4-(Benzyloxy)-2-nitroaniline
	Phenylpropanoids	1-Butanone, 1,4-diphenyl-
	Phenylpropanoids	1,3,5-Triphenyl-1,5-pentanedione
	Linear hydrocarbons	N-heneicosane
	Linear hydrocarbons	Hexacosane
	Linear hydrocarbons	Nonacosane
	Linear hydrocarbons	Tricosane
	Phenylpropanoids	Sakuranetin
	Phenylpropanoids	4',5-Dihydroxy-7-methoxyflavanone
	Phenylpropanoids	Isosakuranetin, diacetate
	Linear hydrocarbons	Tetratriacontane
	Others	14,16-Hentriacontanedione
	Linear hydrocarbons	Nonane, 2,5-dimethyl-
	Terpenoids	cis-linalool oxide
	Terpenoids	Estragole
	Terpenoids	(-)-alpha-fenchol
	Terpenoids	Chavicol
	Linear hydrocarbons	Dodecane, 2-methyl-
	Terpenoids	4,7-Methanoazulene, 1,2,3,4,5,6,7,8-octahydro-1,4,9,9-tetramethyl-
	Phenylpropanoids	Ethanone, 1-(3-hydroxyphenyl)-
	Terpenoids	1-Isopropyl-4,7-dimethyl-1,2,3,4,5,6-hexahydronaphthalene
	Terpenoids	Zonarene
	Terpenoids	Farnesene epoxide, E-
	Terpenoids	2-(4a,8-Dimethyl-2,3,4,5,6,7-hexahydro-1H-naphthalen-2-yl)propan-2-ol
	Terpenoids	Hinesol
	Terpenoids	Isoaromadendrene epoxide
	Terpenoids	(1R,3E,7E,11R)-1,5,5,8-Tetramethyl-12-oxabicyclo[9.1.0]dodeca-3,7-diene
	Terpenoids	(-)-10-epi.gamma.-Eudsemol
GW-4585	Terpenoids	6-Methyl-2-(3-methyl-1-cyclohex-3-enyl)-5-hepten-2-ol
	Terpenoids	2-Naphthalenemethanol, 1,2,3,4,4a,5,6,8a-octahydro-.alpha.,.alpha.,4a,8-tetramethyl-, [2R-(2.alpha.,4a.alpha.,8a.beta.)]-
	Linear hydrocarbons	1-Octadecene
	Phenylpropanoids	Benzoic acid, hept-2-yl ester
	Terpenoids	alpha-sinesal
	Others	1-Hexadecanol, 2-methyl-
	Phenylpropanoids	Phenylethyl salicylate
	Others	1-Methyl-4-(6-methylhept-5-en-2-yl)cyclohexa-1,3-diene
	Phenylpropanoids	piceol
	Phenylpropanoids	4-(Benzyloxy)-3-methoxybenzoic acid
	Linear hydrocarbons	Octadecanoic acid
	Phenylpropanoids	4-(Benzyloxy)-2-nitroaniline
	Phenylpropanoids	5-Hydroxy-4',7-dimethoxyflavanone
	Others	Hexanoic acid, heptadecyl ester
	Linear hydrocarbons	Triacontane, 1-bromo-
	Terpenoids	Squalene
	Linear hydrocarbons	Pentatriacontane
	Linear hydrocarbons	Dodecane
	Linear hydrocarbons	Dodecane, 2,6,11-trimethyl-
	Others	Benzaldehyde, 4-hydroxy-
	Others	1-{3-tricyclo[2.2.1.0(2.6)]heptyl}-1-butanone
	Terpenoids	Naphthalene, 1,2,3,4,4a,7-hexahydro-1,6-dimethyl-4-(1-methylethyl)-
	Terpenoids	Guaiol
	Others	Phenol, 2,4-di-t-butyl-6-nitro-
	Others	3-Methyl-2-butenoic acid, tridec-2-ynyl ester
GW-7096	Others	2,6-Nonadienoic acid, 9-(3,3-dimethyloxiranyl)-3,7-dimethyl-, methyl ester, (E,E)-
	Linear hydrocarbons	1-Tricosene
	Others	Isopropyl palmitate
	Linear hydrocarbons	N-heneicosane
	Phenylpropanoids	Pinostrobin chalcone
	Phenylpropanoids	4H-1-Benzopyran-4-one, 5-hydroxy-7-methoxy-2-phenyl-
	Phenylpropanoids	2',6'-Dihydroxy 4'-methoxydihydrochalcone, diacetate

	Linear hydrocarbons	Nonacosane
	Linear hydrocarbons	Tricosane
	Phenylpropanoids	4',5-Dihydroxy-7-methoxyflavanone
GW-9900	Others	2-Butanone
	Others	2-Cyclohexyl-hex-5-en-2-ol
	Terpenoids	Linalool
	Terpenoids	4-terpineol
	Linear hydrocarbons	Dodecane, 2,6,11-trimethyl-
	Terpenoids	1H-Cycloprop[e]azulene, decahydro-1,1,7-trimethyl-4-methylene-, (1aR,4aS,7R,7aR,7bS)-(-)-
	Terpenoids	(1R,4aS,8aR)-1-Isopropyl-4,7-dimethyl-1,2,4a,5,6,8a-hexahydronaphthalene
	Terpenoids	4.beta.H,5.alpha.-Eremophila-1(10),11-diene
	Terpenoids	Naphthalene, 1,2,4a,5,6,8a-hexahydro-4,7-dimethyl-1-(1-methylethyl)-, [1S-(1.alpha.,4a.beta.,8a.alpha.)]-
	Terpenoids	.alpha.-Muurolene
	Terpenoids	Naphthalene, 1,2,3,4,4a,5,6,8a-octahydro-7-methyl-4-methylene-1-(1-methylethyl)-, (1.alpha.,4a.beta.,8a.alpha.)-
	Terpenoids	Naphthalene, 1,2,3,4,4a,7-hexahydro-1,6-dimethyl-4-(1-methylethyl)-
	Terpenoids	(-)-1,2,2.alpha.,3,3,4,6,7,8,8.alpha.-decahydro-2.alpha.,7,8-trimethylacenaphthylene
	Terpenoids	1,6,10-Dodecatrien-3-ol, 3,7,11-trimethyl-, (E)-
	Terpenoids	.tau.-Cadinol
	Terpenoids	2-Naphthalenemethanol, 1,2,3,4,4a,5,6,7-octahydro-.alpha.,.alpha.,4a,8-tetramethyl-, (2R-cis)-
	Terpenoids	Isoaromadendrene epoxide
	Linear hydrocarbons	1-Octadecene
	Phenylpropanoids	Benzoic acid, 1-phenylethyl ester
	Terpenoids	[(1S,7S,8S,8aS)-8-[2-[(2R,4R)-4-Hydroxy-6-oxooxan-2-yl]ethyl]-7-methyl-1,2,3,4,4a,7,8,8a-octahydronaphthalen-1-yl] (2S)-2-methy
	Linear hydrocarbons	Isoheptadecanol
	Phenylpropanoids	2-Methoxybenzoic acid, benzyl ester
	Phenylpropanoids	3-Phenylpropionyl fluoride-2,2-D2
	Linear hydrocarbons	N-heneicosane
	Linear hydrocarbons	Tetracosane
	Linear hydrocarbons	2-Methylhexacosane
	Linear hydrocarbons	Hexatriacontane
	Phenylpropanoids	2,2,5-trimethyl-5-(3-methylbut-2-enyl)-8-oxidanyl-7-(3-phenylpropanoyl)chromen-6-one
HOPF-27-3	Others	1,3,5,7-Cyclooctatetraene
	Terpenoids	2-Pinene
	Linear hydrocarbons	Decane
	Linear hydrocarbons	Undecane
	Linear hydrocarbons	Nonane, 2,5-dimethyl-
	Terpenoids	Linalool
	Terpenoids	(1S)-1,3,3-trimethylnorbornan-2-ol
	Terpenoids	4-terpineol
	Terpenoids	Beta-cyclocitral
	Terpenoids	beta-cyclocitral
	Terpenoids	Chavicol
	Others	Benzaldehyde, 4-hydroxy-
	Linear hydrocarbons	Hexadecane
	Phenylpropanoids	2-Propenoic acid, 3-phenyl-
	Terpenoids	Naphthalene, decahydro-4a-methyl-1-methylene-7-(1-methylethenyl)-, [4aR-(4a.alpha.,7.alpha.,8a.beta.)]-
	Terpenoids	4.beta.H,5.alpha.-Eremophila-1(10),11-diene
	Terpenoids	.alpha.-Muurolene
	Terpenoids	trans-.alpha.-Bergamotene
	Terpenoids	1,6,10-Dodecatrien-3-ol, 3,7,11-trimethyl-, (E)-
	Terpenoids	gamma-ionone
	Terpenoids	4,11-Dimethyl-8-(propan-2-yl)-5,12-dioxatricyclo[9.1.0.04,6]dodecan-7-ol, Ac
	Terpenoids	2-Adamantanol, 2-(bromomethyl)-
	Terpenoids	4a(2H)-Naphthalenol, 1,3,4,5,6,8a-hexahydro-4,7-dimethyl-1-(1-methylethyl)-, (1S,4S,4aS,8aR)-
	Terpenoids	trans-Z-.alpha.-Bisabolene epoxide
	Terpenoids	1-Naphthalenol, 1,2,3,4,4a,7,8,8a-octahydro-1,6-dimethyl-4-(1-methylethyl)-, [1R-(1.alpha.,4.beta.,4a.beta.,8a.beta.)]-
	Terpenoids	Agarospirol
	Terpenoids	.alpha.-Cadinol
	Phenylpropanoids	Phenol, 2,4-di-t-butyl-6-nitro-
	Terpenoids	3-Cyclohexene-1-methanol, .alpha.,4-dimethyl-.alpha.-(4-methyl-3-penten
	Phenylpropanoids	Benzene, 1,1'-[1-(2,2-dimethyl-3-butenyl)-1,3-propanediyl]bis-
	Phenylpropanoids	Benzoic acid, 1-phenylethyl ester
	Linear hydrocarbons	Octadecane, 1-chloro-
	Terpenoids	[(1S,7S,8S,8aS)-8-[2-[(2R,4R)-4-Hydroxy-6-oxooxan-2-yl]ethyl]-7-methyl-1,2,3,4,4a,7,8,8a-octahydronaphthalen-1-yl] (2S)-2-methy
	Terpenoids	alpha-sinesal
	Phenylpropanoids	2,2,5-trimethyl-5-(3-methylbut-2-enyl)-8-oxidanyl-7-(3-phenylpropanoyl)chromen-6-one
	Others	9,12-Octadecadienoic acid (Z,Z)-, methyl ester
	Phenylpropanoids	2-Methoxybenzoic acid, 2-phenylethyl ester
	Phenylpropanoids	3-Phenylpropionyl fluoride-2,2-D2
	Phenylpropanoids	4-(Benzyloxy)-3-methoxybenzoic acid
	Linear hydrocarbons	Octadecanoic acid
	Linear hydrocarbons	Eicosane
	Linear hydrocarbons	Hexacosane
	Linear hydrocarbons	Tetracosane
	Phenylpropanoids	Pinostrobin chalcone
	Linear hydrocarbons	Hexatriacontane
	Phenylpropanoids	2',4',6'-Trihydroxydihydrochalcone
	Linear hydrocarbons	Tetratriacontane
	Linear hydrocarbons	Triacontane, 1-bromo-
	Terpenoids	2-Pinene
	Linear hydrocarbons	Decane
	Others	D-Limonene
	Others	Nonanal
	Phenylpropanoids	4-Vinylphenol
	Terpenoids	(3R,6S)-2,2,6-Trimethyl-6-vinyltetrahydro-2H-pyran-3-ol
	Linear hydrocarbons	Tetradecane
	Phenylpropanoids	2-Propenoic acid, 3-phenyl-
	Terpenoids	trans-.alpha.-Bergamotene
	Linear hydrocarbons	1-Octadecanesulphonyl chloride
	Others	10-12-Pentacosadiynoic acid

	Others	Phenol, 2,4-di-t-butyl-6-nitro-
	Terpenoids	4-(2,2,6-Trimethylcyclohexyl)-2-butanone
	Linear hydrocarbons	Octadecane, 1-chloro-
	Terpenoids	alpha-sinesal
	Others	2-Acetyl-1-pyrroline
	Others	2-(2,2-Dimethylpropanoyl)cyclohexanone
	Linear hydrocarbons	Isoheptadecanol
	Others	1-Nonadecene
	Linear hydrocarbons	1-Tricosene
KTMA-12-2	Phenylpropanoids	4-Methoxybenzyl benzoate
	Linear hydrocarbons	Octadecanoic acid
	Phenylpropanoids	Benzenemethanol, 2-hydroxy-3,6-dimethyl-4-(phenylmethoxy)-
	Phenylpropanoids	1,3,5-Triphenyl-1,5-pentanedione
	Linear hydrocarbons	Eicosane
	Others	9-Octadecenamide
	Linear hydrocarbons	N-heneicosane
	Linear hydrocarbons	Hexacosane
	Phenylpropanoids	2-Butanone, 4-(4-hydroxyphenyl)-
	Phenylpropanoids	Cinnamyl cinnamate
	Linear hydrocarbons	2-Methylhexacosane
	Phenylpropanoids	4H-1-Benzopyran-4-one, 5-hydroxy-7-methoxy-2-phenyl-
	Others	4H-1-Benzopyran-4-one, 5,7-dihydroxy-2-phenyl-
	Linear hydrocarbons	Nonacosane
	Phenylpropanoids	2,2,5-trimethyl-5-(3-methylbut-2-enyl)-8-oxidanyl-7-(3-phenylpropanoyl)chromen-6-one
	Phenylpropanoids	Naphthalene, 1,2,3,4-tetrahydro-1-phenyl-
	Phenylpropanoids	4H-1-Benzopyran-4-one, 2,3-dihydro-5-hydroxy-2-(4-hydroxyphenyl)-7-methoxy-, (S)-
	Linear hydrocarbons	Tetratriacontane
	Linear hydrocarbons	Triacontane, 1-bromo-
	Others	14,16-Hentriacontanedione
	Linear hydrocarbons	Pentatriacontane
	Others	Eicosanoic acid, 2,3-bis(acetyloxy)propyl ester
	Others	Butanoic acid, 3-methyl-, ethyl ester
	Linear hydrocarbons	Decane
	Terpenoids	4-terpineol
	Linear hydrocarbons	Dodecane, 2,6,11-trimethyl-
	Terpenoids	Beta-cyclocitral
	Phenylpropanoids	4-Vinylphenol
	Linear hydrocarbons	Dodecane, 2-methyl-
	Linear hydrocarbons	Tetradecane
	Linear hydrocarbons	Hexadecane
	Phenylpropanoids	2-Propenoic acid, 3-phenyl-
	Phenylpropanoids	Ethanone, 1-(2-hydroxyphenyl)-
	Terpenoids	(1R,4aS,8aR)-1-Isopropyl-4,7-dimethyl-1,2,4a,5,6,8a-hexahydronaphthalene
	Linear hydrocarbons	1-Octadecanesulphonyl chloride
	Phenylpropanoids	Benzene, 1,1'-[1-(2,2-dimethyl-3-butenyl)-1,3-propanediyl]bis-
	Phenylpropanoids	Benzoic acid, hept-2-yl ester
	Terpenoids	2-Cyclohexen-1-one, 2-methyl-5-(1-methylethenyl)-
NHTA-27-3	Phenylpropanoids	Benzoic acid, 2-hydroxy-, phenylmethyl ester
	Phenylpropanoids	Phenylethyl salicylate
	Phenylpropanoids	(E)-Cinnamyl benzoate
	Phenylpropanoids	Benzoic acid, 2-methoxy-, methyl ester
	Phenylpropanoids	2-Methoxybenzoic acid, 2-phenylethyl ester
	Phenylpropanoids	piceol
	Phenylpropanoids	4-(Benzyloxy)-3-methoxybenzoic acid
	Phenylpropanoids	Benzenemethanol, 2-hydroxy-3,6-dimethyl-4-(phenylmethoxy)-
	Linear hydrocarbons	N-heneicosane
	Linear hydrocarbons	Tetracosane
	Phenylpropanoids	2-Propen-1-one, 1-(2,6-dihydroxy-4-methoxyphenyl)-3-phenyl-, (E)-
	Phenylpropanoids	pinostrobin chalcone
	Phenylpropanoids	5-Hydroxy-4',7-dimethoxyflavanone
	Phenylpropanoids	Naphthalene, 1,2,3,4-tetrahydro-1-phenyl-
	Others	Ethanone, 1-[2,3-dihydro-6-hydroxy-2-(1-hydroxy-1-methylethyl)-4-methoxy-7-benzofuranyl]-, (+)-
	Linear hydrocarbons	Triacontane, 1-bromo-
	Terpenoids	Squalene
	Linear hydrocarbons	Undecane
	Linear hydrocarbons	Nonane, 2,5-dimethyl-
	Terpenoids	(3R,6S)-2,2,6-Trimethyl-6-vinyltetrahydro-2H-pyran-3-ol
	Linear hydrocarbons	Dodecane
	Phenylpropanoids	4-Vinylphenol
	Linear hydrocarbons	Dodecane, 2-methyl-
	Linear hydrocarbons	Hexadecane
	Phenylpropanoids	Acetophenone, 4'-hydroxy-
	Phenylpropanoids	Ethanone, 1-(2-hydroxyphenyl)-
	Phenylpropanoids	Ethanone, 1-(3-hydroxyphenyl)-
	Terpenoids	(1R,4aS,8aR)-1-Isopropyl-4,7-dimethyl-1,2,4a,5,6,8a-hexahydronaphthalene
	Others	2,4-Di-tert-butylphenol
	Linear hydrocarbons	1-Octadecanesulphonyl chloride
	Others	Acetic acid, 3-hydroxy-6-isopropenyl-4,8a-dimethyl-1,2,3,5,6,7,8,8a-octahydronaphthalen-2-yl ester
	Terpenoids	trans-Z-.alpha.-Bisabolene epoxide
	Terpenoids	1-Naphthalenol, 1,2,3,4,4a,7,8,8a-octahydro-1,6-dimethyl-4-(1-methylethyl)-, [1R-(1.alpha.,4.beta.,4a.beta.,8a.beta.)]-
	Terpenoids	Guaiol
	Terpenoids	2-Naphthalenemethanol, decahydro-.alpha.,.alpha.,4a-trimethyl-8-methylene-, [2R-(2.alpha.,4a.alpha.,8a.beta.)]-
	Phenylpropanoids	[3,3-Dimethyl-1-(2-phenylethyl)-4-pentenyl]benzene
	Phenylpropanoids	4-Pentenoic acid, 5-phenyl-
	Terpenoids	4-(2,2,6-Trimethylcyclohexyl)-2-butanone
	Linear hydrocarbons	1-Octadecene
	Phenylpropanoids	3-Hexen-1-ol benzoate
	Terpenoids	cryptomeridiol
WELC-27-4	Others	1-Hexadecanol, 2-methyl-
	Others	1-Nonadecene

	Phenylpropanoids	Phenylethyl salicylate
	Phenylpropanoids	4-Methoxybenzyl benzoate
	Phenylpropanoids	2-Methoxybenzyl benzoate
	Phenylpropanoids	Benzoic acid, 2-methoxy-, methyl ester
	Phenylpropanoids	2-Methoxybenzoic acid, 2-phenylethyl ester
	Phenylpropanoids	2-Phenylpropionsaeure
	Phenylpropanoids	Benzoic acid, 2-hydroxy-4-[(6-hydroxy-3,4-dimethoxy-2-methylbenzoyl)oxy]-6-methyl-, 3-hydroxy-5-methyl-4-[(phenylmethoxy)carbon
	Phenylpropanoids	piceol
	Linear hydrocarbons	Octadecanoic acid
	Phenylpropanoids	1,3,5-Triphenyl-1,5-pentanedione
	Linear hydrocarbons	Eicosane
	Linear hydrocarbons	N-heneicosane
	Phenylpropanoids	(-)-Pinostrobin
	Phenylpropanoids	Pinostrobin chalcone
	Phenylpropanoids	Cinnamyl cinnamate
	Terpenoids	4H-1-Benzopyran-4-one, 2,3-dihydro-5,7-dihydroxy-2-phenyl-, (S)-
	Phenylpropanoids	Benzofuran-6-ol-3-one, 2-(4-ethoxycarbonyl)benzylidene-
	Linear hydrocarbons	Tricosane
	Phenylpropanoids	8-Hydroxy-2,2,5-trimethyl-5-(3-methylbut-2-en-1-yl)-7-(3-phenylpropanoyl)-2H-chromen-6(5H)-one
	Phenylpropanoids	5-Hydroxy-4',7-dimethoxyflavanone
	Phenylpropanoids	(E)-hinokiresinol
	Phenylpropanoids	4',5-Dihydroxy-7-methoxyflavanone
	Linear hydrocarbons	Triacontane, 1-bromo-
	Linear hydrocarbons	Pentatriacontane
	Others	Butanoic acid, 2-methyl-, ethyl ester
	Phenylpropanoids	Benzene, 1,3-dimethyl-
	Terpenoid	2-Pinene
	Linear hydrocarbons	Decane
	Terpenoid	D-Limonene
	Others	2-Pyrrolidinone, 1-(2-aminoethyl)-
	Terpenoid	Linalool
	Others	Nonanal
	Terpenoid	.alpha.-Terpineol
	Linear hydrocarbons	Dodecane
	Linear hydrocarbons	Tetradecane
	Others	Acetophenone, 4'-hydroxy-
	Others	2,4-Di-tert-butylphenol
NHTA-27-5	Terpenoid	1,6,10-Dodecatrien-3-ol, 3,7,11-trimethyl-, (E)-
	Terpenoid	Bergamotol, Z-.alpha.-trans-
	Others	Phenol, 2,4-di-t-butyl-6-nitro-
	Phenylpropanoids	Benzyl Benzoate
	Phenylpropanoids	Benzoic acid, 1-phenylethyl ester
	Phenylpropanoids	3-Hexen-1-ol benzoate
	Phenylpropanoids	Benzoic acid, 2-hydroxy-, phenylmethyl ester
	Phenylpropanoids	2-Methoxybenzoic acid, benzyl ester
	Linear hydrocarbons	N-heneicosane
	Linear hydrocarbons	Eicosane
	Phenylpropanoids	pinostrobin chalcone
	Linear hydrocarbons	Nonacosane
	Phenylpropanoids	2',6'-Dihydroxy 4'-methoxydihydrochalcone, diacetate
	Phenylpropanoids	Benzenepropanoic acid, 3-phenyl-2-propenyl ester
	Phenylpropanoids	5-Hydroxy-4',7-dimethoxyflavanone
